# Mitochondrial and Plastid Genomes from Coralline Red Algae Provide Insights into the Incongruent Evolutionary Histories of Organelles

**DOI:** 10.1093/gbe/evy222

**Published:** 2018-10-26

**Authors:** Jun Mo Lee, Hae Jung Song, Seung In Park, Yu Min Lee, So Young Jeong, Tae Oh Cho, Ji Hee Kim, Han-Gu Choi, Chang Geun Choi, Wendy A Nelson, Suzanne Fredericq, Debashish Bhattacharya, Hwan Su Yoon

**Affiliations:** 1 Department of Biological Sciences, Sungkyunkwan University, Suwon, Korea; 2 Department of Marine Life Science, Chosun University, Gwangju, Korea; 3 Division of Life Sciences, Korea Polar Research Institute, KOPRI, Incheon, Korea; 4 Department of Ecological Engineering, Pukyong National University, Busan, Korea; 5 National Institute for Water and Atmospheric Research, Wellington, New Zealand; 6 School of Biological Sciences, University of Auckland, New Zealand; 7 Biology Department, University of Louisiana at Lafayette, Lafayette, Louisiana; 8 Department of Biochemistry and Microbiology, Rutgers University

**Keywords:** organelle genome evolution, coralline red algae, Rhodophyta

## Abstract

Mitochondria and plastids are generally uniparentally inherited and have a conserved gene content over hundreds of millions of years, which makes them potentially useful phylogenetic markers. Organelle single gene-based trees have long been the basis for elucidating interspecies relationships that inform taxonomy. More recently, high-throughput genome sequencing has enabled the construction of massive organelle genome databases from diverse eukaryotes, and these have been used to infer species relationships in deep evolutionary time. Here, we test the idea that despite their expected utility, conflicting phylogenetic signal may exist in mitochondrial and plastid genomes from the anciently diverged coralline red algae (Rhodophyta). We generated complete organelle genome data from five coralline red algae (*Lithothamnion* sp., *Neogoniolithon spectabile*, *Renouxia* sp., *Rhodogorgon* sp., and *Synarthrophyton chejuensis*) for comparative analysis with existing organelle genome data from two other species (*Calliarthron tuberculosum* and *Sporolithon durum*). We find strong evidence for incongruent phylogenetic signal from both organelle genomes that may be explained by incomplete lineage sorting that has maintained anciently derived gene copies or other molecular evolutionary processes such as hybridization or gene flow during the evolutionary history of coralline red algae.

## Introduction

Mitochondria and plastids originated from prokaryotes (i.e., α-proteobacteria and cyanobacteria, respectively) through independent primary endosymbioses that occurred early in eukaryote evolution, over a billion years ago (Timmis et al. 2004). These prokaryotic symbionts were integrated as intracellular compartments in the eukaryotic host cell through the process of organellogenesis, whereby their genomes were reduced due to outright gene loss or by endosymbiotic gene transfer (EGT) to the nuclear genome (Martin and Herrmann 1998; Bhattacharya et al. 2004; Timmis et al. 2004). Organelles are essential for several important metabolic pathways involved in photosynthesis and energy cycles in eukaryotic cells (Martin and Schnarrenberger 1997; Martin and Herrmann 1998; Saraste 1999; Burger et al. 2003; Herrmann 2003). Through a series of endosymbioses involving different host cells, about ten diverse phyla (e.g., Euglenophyta, Heterokontophyta, Haptophyta, Dinozoa) became plastid-bearing photosynthetic eukaryotes. The members of most of these lineages share a common evolutionary history even if they contain three genomes from two organelles and one host. Because of a conserved gene content and low mutation rates, when compared with nuclear genes, organelle genome data have been frequently applied to evolutionary studies. High-throughput DNA sequencing has empowered the construction of massive organelle genome databases from diverse eukaryotes that has resulted in many new insights into their phylogenetic relationships and the molecular evolution of the organelle genomes (Smith and Keeling 2015). For example, based on mitochondrial and plastid genome data, red algal phylogenetic relationships have been robustly reconstructed (Yang et al. 2015; Lee, Cho, et al. 2016; Muñoz-Gómez et al. 2017).

Despite the rich phylogenetic information, incongruent phylogenetic relationships between organelle (mitochondria or plastid) and nuclear genes were recently revealed. For example, it was found that the plastid (*trn*T–*trn*L, *mat*K) and nuclear (ITS) sequence data from a flowering plant, *Pilosella* hawkweeds, contain phylogenetic incongruence derived from ancient intergeneric hybridization (Fehrer et al. 2007). An insect, the Hawaiian cricket, also showed incongruence between mitochondrial and nuclear DNA phylogenies (Shaw 2002). Phylogenies of the mitochondrial genome, Y chromosome, and nuclear genome fragments of bears showed incongruence caused by introgression and gene flow (Kutschera et al. 2014; Kumar et al. 2017). Such results have been frequently reported from animal groups (Toews and Brelsford 2012), including a genome-wide analysis of birds that uncovered incomplete lineage sorting (ILS) caused by a rapid radiation, particularly in early-diverged lineages (Jarvis et al 2014). In the red algae (Rhodophyta), phylogenetic incongruence was reported between two early diverged coralline species based on plastid (*psb*A) and nuclear (18S rRNA) markers (Broom et al. 2008). In the calcified red algal group Corallinophycidae, phylogenetic analyses using molecular markers are necessary because these species display complex morphological diversity including geniculate (branching), nongeniculate (crustose), and rhodolith (free-living) forms ( as well as convergent morphologies within these growth forms; Adey and Macintyre 1973; Bosence 1983; Foster 2001). However, complete organelle genomes have been reported for only two species within this subclass (Janouškovec et al. 2013; Lee, Cho, et al. 2016).

To better understand organelle genome evolution and inspect the phylogenetic signal encoded by mitochondrial and plastid genes, we generated a total of 10 complete organelle genomes (five plastids and five mitochondria) from five coralline species (*Lithothamnion* sp., *Neogoniolithon spectabile*, *Renouxia* sp., *Rhodogorgon* sp., and *Synarthrophyton chejuensis*). These data were then compared with existing organelle genomes from two coralline red algae (*Calliarthron tuberculosum* and *Sporolithon durum*). These seven genomes encompass the four major orders (Sporolithales, Rhodogorgonales, Hapalidiales, and Corallinales) of the subclass Corallinophycidae (class Florideophyceae; Le Gall and Saunders 2007; Nelson et al. 2015). From these analyses, we report incongruent phylogenetic histories between mitochondrial and plastid genome data between the order Sporolithales and Rhodogorgonales. Because phylogenetic incongruence between mitochondrial and plastid trees and between 18S and 28S rRNA trees was unexpected, we describe here these major trends in the genealogical history of organelle genomes and discuss how to interpret these incongruent phylogenetic signals in the coralline algae.

## Materials and Methods

### Genome Sequencing, Assembly, Gene Prediction and Annotation

Samples of coralline red algal species *Lithothamnion* sp. (LAF6882; Campeche Banks, Mexico, SW Gulf of Mexico, coll. S. Fredericq), *N.spectabile* (LAF6908A; Apr. 19, 2014, Florida Keys, Florida, USA, coll. S. Fredericq), *Renouxia* sp. (LAF6170; May 13, 2012, Hurghada, Egypt, coll. Thomas Sauvage), *Rhodogorgon* sp. (SGAD1304047; Dec. 15, 2013, Ternate Island, Nusa Tenggara Timur, Indonesia, coll. S. G. Draisma), and *Syn.chejuensis* (Sep. 20, 2015, Song-do beach, Busan, Korea) were collected from the subtidal zone. Genomic DNA was extracted using the DNeasy Plant Mini Kit (Qiagen, Hilden, Germany). These coralline species were identified based on morphological features as well as phylogenetic analyses using the *psb*A and *cox*1 genes (supplementary fig. S1, Supplementary Material online). However, three species (*Lithothamnion*, *Renouxia*, and *Rhodogorgon* species) were not resolved at the species-level due to a lack of authentic sequences from type material. The following species were chosen for analysis: a rhodolith-forming taxon (i.e., *Lithothamnion* sp.), two nongeniculate species (*N.spectabile*, *Syn.chejuensis*), and two fleshy species (*Renouxia* sp. and *Rhodogorgon* sp.). The coralline taxa were subsampled with care to avoid contamination; therefore, it is unlikely (though not impossible) that the selected samples were contaminated by different species in their natural habitats as may be the case with crustose species where one species may grow on top of another crust. The HiSeq2000 sequencing platform (Illumina, San Diego) was applied to generate genome sequencing data of *Syn.chejuensis* using 100 bp paired-end sequencing library. Other coralline genome sequencing data were generated using the Ion Torrent PGM platform (Thermo Fisher Scientific, San Francisco, California) with 400 bp-sized sequencing libraries. To check for cross contamination, or mixed samples, several molecular markers (i.e., 18S rRNA, *rbc*L, and *psb*A) were identified in the assembled genome data, and their phylogenetic analysis confirmed that there were no mixed coralline species in each data set (i.e., the single, expected marker was found). Organelle genome assemblies and annotations followed Song et al. (2016). The sequenced raw reads were assembled using the CLC Genomics Workbench 5.5.1 (CLC bio, Aarhus, Denmark) and MIRA assemblers. Contigs of organelle genome were sorted by customized Python scripts with a local BLAST program and re-assembled to construct consensus genome sequences. Draft organelle genomes were confirmed using the read-mapping method in CLC Genomics Workbench 5.5.1. Gap sequences were verified by PCR-based Sanger sequencing.

Organelle gene prediction and annotation were done using Geneious 8.1.2 (Kearse et al. 2012) based on BlastX search results (*e*-value ≤ 1.0*e*^−05^) with codon table 4 (The Mold, Protozoan, and Coelenterate Mitochondrial Code and the Mycoplasma/Spiroplasma Code) and codon table 11 (Bacterial, Archaeal and Plant Plastid Code). Ribosomal RNAs (rRNAs) and transfer RNAs (tRNAs) were predicted by the web-based programs RNAmmer 1.2 Server and ARAGORN (Laslett and Canback 2004; Lagesen et al. 2007). RNAweasel (http://megasun.bch.umontreal.ca/cgi-bin/RNAweasel/RNAweaselInterface.pl) was used to predict group II introns. The 18S and 28S rRNA regions were sorted from the genome assembly of each sequencing data using BlastN search (*e*-value ≥ 1.*e*^−20^) and then the full length was confirmed using the web-based RNAmmer 1.2 program (Lagesen et al. 2007).

### Comparison of Organelle Genome Structure and Phylogenetic Analysis

Structures of organelle genomes were compared using MAUVE 2.3.1 (Darling et al. 2004) with “default options”. To construct maximum likelihood (ML) trees, organelle coding genes were aligned using MAFFT 7.110 under default settings (Katoh and Toh 2008) and ML trees were constructed using IQ tree (Minh et al. 2013; Flouri et al. 2015; Nguyen et al. 2015) with the predicted amino acid sequences. The phylogenetic model was chosen through the model test option (-m TEST), followed by the ML tree search, and ultrafast bootstrapping with 1,000 replications (-bb 1,000). Concatenated alignments of 22 mitochondrial and 195 plastid genes were constructed using a customized Python script and then analyzed with the gene partition information (-q). The approximately unbiased test (AU test) was done by IQ tree with the possible tree topologies (-z topology.treefile -zb 1000 -au). To sort subtopologies from constructed ML trees, the PyCogent python module was used and the results were manually confirmed (https://github.com/pycogent/pycogent). Several representative ML trees were merged by the method of intertwining phylogenetic networks (Schliep et al. 2017) and visualized with the phangorn package in R (https://github.com/KlausVigo/phangorn).

## Results and Discussion

### General Features of Coralline Mitochondrial Genomes

Mitochondrial genomes (mtDNAs) of five coralline species were assembled using high-throughput sequencing data from *Renouxia* sp. (1.5 Gbp; Ion Torrent PGM), *Rhodogorgon* sp. (1.5 Gbp; Ion Torrent PGM), *Lithothamnion* sp. (882 Mbp; Ion Torrent PGM), *N.spectabile* (972 Mbp; Ion Torrent PGM), and *Syn.chejuensis* (18 Gbp; Illumina HiSeq2000). The mtDNAs of *Renouxia* sp. (30,019 bp, GC: 27.0%) and *Rhodogorgon* sp. (30,547 bp, GC: 26.0%) were circa 2–5 kbp larger than those of *Lithothamnion* sp. (25,605 bp, GC: 27.2%), *Syn. chejuensis* (28,264 bp, GC: 25.2%) and *N.spectabile* (26,050 bp, GC: 29.6%) as well as two published coralline mtDNAs *Spo.durum* (26,202 bp, GC: 28.4%), and *C.tuberculosum* (26,469 bp, GC: 27.3%; supplementary table S1, Supplementary Material online; Bi et al. 2015; Kim et al. 2015). The structures of these coralline mtDNAs were conserved with some size variation (ranging from 25 to 30 kbp; supplementary table S1 and fig. S2, Supplementary Material online), and the conserved structure was also observed in the sister taxa of Nemaliophycidae (supplementary fig. S2, Supplementary Material online; Yang et al. 2015). The mtDNAs of most coralline species contain two rRNAs and around 25 protein-coding sequences, except the mtDNA of *N.spectabile* that showed pseudogenization of several conserved CDSs (e.g., *atp*8, *rpl*20, and *sdh*4). Nineteen to twenty-five tRNAs were commonly found between the *sec*Y and *atp*6 genes as in other red algal mtDNAs (Lee et al. 2015; Yang et al. 2015). All seven coralline species encoded a group II intron-containing tRNA (trnI) between the *nad*5 and *nad*4 genes in mtDNA (supplementary table S2, Supplementary Material online).

It is notable that two Rhodogorgonales species (*Renouxia* sp. and *Rhodogorgon* sp.) contained additional introns in the *cox*1 (two introns with intronic *orf*780 and *orf*790) and *rrl* (one intron) regions. The total sequence lengths of these introns were 4,875 bp in *Renouxia* sp. (*cox*1: 463 + 3,794 bp and *rrl*: 618 bp) and 4,774 bp in *Rhodogorgon* sp. (*cox*1: 491 + 3,658 bp and *rrl*: 625 bp). These introns and intronic ORFs were one of the major contributors to size variation, together with noncoding regions (*Spo. durum*: 4,626 bp, *Renouxia* sp.: 6,139 bp, *Rhodogorgon* sp.: 6,188 bp, *Lithothamnion* sp.: 3,260 bp, *Syn. chejuensis*: 6,050 bp, *C. tuberculosum*: 4,220 bp, and *N.spectabile*: 5,552 bp). The homologs of Rhodogorgonales *orf*780 gene were also found in various eukaryotes (i.e., rhodophytes, Viridiplantae, stramenopiles, fungi, and cryptophytes), but prokaryotic homologs were not identified from the public database (blastp *e*-value ≥ 1.*e*^−05^ to local RefSeq database; supplementary fig. S3, Supplementary Material online). On the basis of the Conserved Domain search (Marchler-Bauer et al. 2017), most of these homologous genes encode group II intron-derived reverse transcriptase domain superfamily members (Intron_maturas2 domain superfamily, cl03174). Homologs of the Rhodogorgonales *orf*790 gene were also distributed in the mtDNAs of various eukaryotes; however, this gene showed a close relationship to diverse prokaryotic lineages (e.g., Proteobacteria, Bacteroidetes, Cyanobacteria, Chloroflexi, Firmicutes) as well as with plastid-encoded genes (e.g., Viridiplantae, cryptophytes, and euglenophytes; supplementary fig. S4, Supplementary Material online). On the basis of this result, we postulate that *orf*790 originated from endosymbiotic prokaryotes and then spread into eukaryotic organelle genomes. Only Rhodogorgonales contains the *orf*790 gene among seven coralline mtDNAs, but this gene is found in other red algae including *Ahnfeltia plicata* (class Florideophyceae; subclass Ahnfeltiophycidae) and Bangiophyceae species (genus *Bangia*, *Porphyra*, and *Pyropia*). Although most eukaryote copies contain the reverse transcriptase domain superfamilies (RVT_1, cl26764 and RVT_N, cl16337; Marchler-Bauer et al. 2017), these other red algal *orf*790 homologs did not form a monophyletic group (supplementary fig. S4, Supplementary Material online). The means and timing of spread of *orf*790 homologs in eukaryotes are still unclear, however, one possible scenario might be due to opportunistic gene transfer from prokaryotes into organelle genomes through a genetic vector (e.g., plasmid). This is because plasmid-mediated horizontal gene transfers have been frequently observed in red algal organelle genomes (Lee, Kim, et al. 2016).

### General Features of Coralline Plastid Genomes

The plastid genomes (ptDNAs) of *Renouxia* sp. (192,307 bp, GC: 32.8%), *Rhodogorgon* sp. (190,860 bp, GC: 32.9%) and *Spo.durum* (191,464 bp, GC: 29.3%) were larger than those of *Lithothamnion* sp. (183,822 bp, GC: 31.1%), *Syn.chejuensis* (179,264 bp, GC: 28.8%), *N.spectabile* (174,280 bp, GC: 33.4%), and *C.tuberculosum* ( 178,981 bp, GC: 29.2%; supplementary table S1, Supplementary Material online; Janouškovec et al. 2013; Lee, Cho, et al. 2016). The structures of these coralline ptDNAs were conserved with some minor size variation (192–174 kbp; supplementary table S1 and fig. S5, Supplementary Material online). The noncoding sequences of coralline ptDNAs also contributed to the size variation as found in mtDNAs (*Spo. durum*: 42,401 bp, *Renouxia* sp.: 35,859 bp, *Rhodogorgon* sp.: 36,530 bp, *Lithothamnion* sp.: 31,315 bp, *Syn. chejuensis*: 24,945 bp, *C. tuberculosum*: 25,675 bp, and *N.spectabile*: 24,845 bp). The ptDNAs of coralline species contained ∼200 protein coding regions (CDSs), 30 tRNAs, three rRNAs, and two intron sequences (trnMe tRNA and *chl*B gene; supplementary table S1, Supplementary Material online) that these contents compositions of ptDNAs were typical in early diverged red algal subclasses (i.e., Corallinophycidae, Nemaliophycidae, and Hildenbrandiophycidae; Lee, Cho, et al. 2016).

We found, however, an unusual 7 kbp insertion in the *Renouxia* sp. ptDNA located between the *rpl*19 and *ilv*B genes that included several plasmid-mediated gene transfers (six *orf*s and one pseudogenized *orf*; supplementary table S3, Supplementary Material online). A similar region was found in the Gracilariales species (subclass Rhodymeniophycidae), which is a distantly related red algal order (Lee, Cho, et al. 2016). These plasmid-related sequences were frequently found in red algal organelle genomes and are likely derived from the plasmid itself or from foreign genetic materials encoded on the plasmid (Lee, Kim, et al. 2016). Conspicuously, there were two novel transferred genes located in the ptDNA of *Renouxia* sp. One of these hypothetical proteins is clustered with the bacterial cupin domain containing proteins (blastp results; *e*-value ≥ 1.*e*^−05^ to NCBI nr database) in the ML tree (supplementary fig. S6*A*, Supplementary Material online; Conserved Domain search; Marchler-Bauer et al. 2017). The other hypothetical protein in *Renouxia* sp. is griffithsin (synthetic protein)-like protein that is clustered with the bacterial jacalin-related lectin protein and its homologs (domain code: cl03205) from diverse bacteria including several cyanobacterial species (supplementary fig. S6*B*, Supplementary Material online). The jacalin-like lectins are sugar-binding protein domains that are mostly found in land plants (Peumans et al. 2001), however, there were no land plant homologs in the blastp search in this study of the griffithsin-like protein (*e*-value ≥ 1.*e*^−05^ to NCBI nr database). We postulate that these two hypothetical proteins in ptDNA of *Renouxia* sp. were independently transferred from bacteria and were mediated by red algal plasmids. It is likely that this mobile element (i.e., plasmid) plays a key role in the acquisition of foreign genes (i.e., novel genetic resources) and thereby, organelle genome evolution.

### Phylogenetic Analyses of Coralline Species

**Fig. 1.**
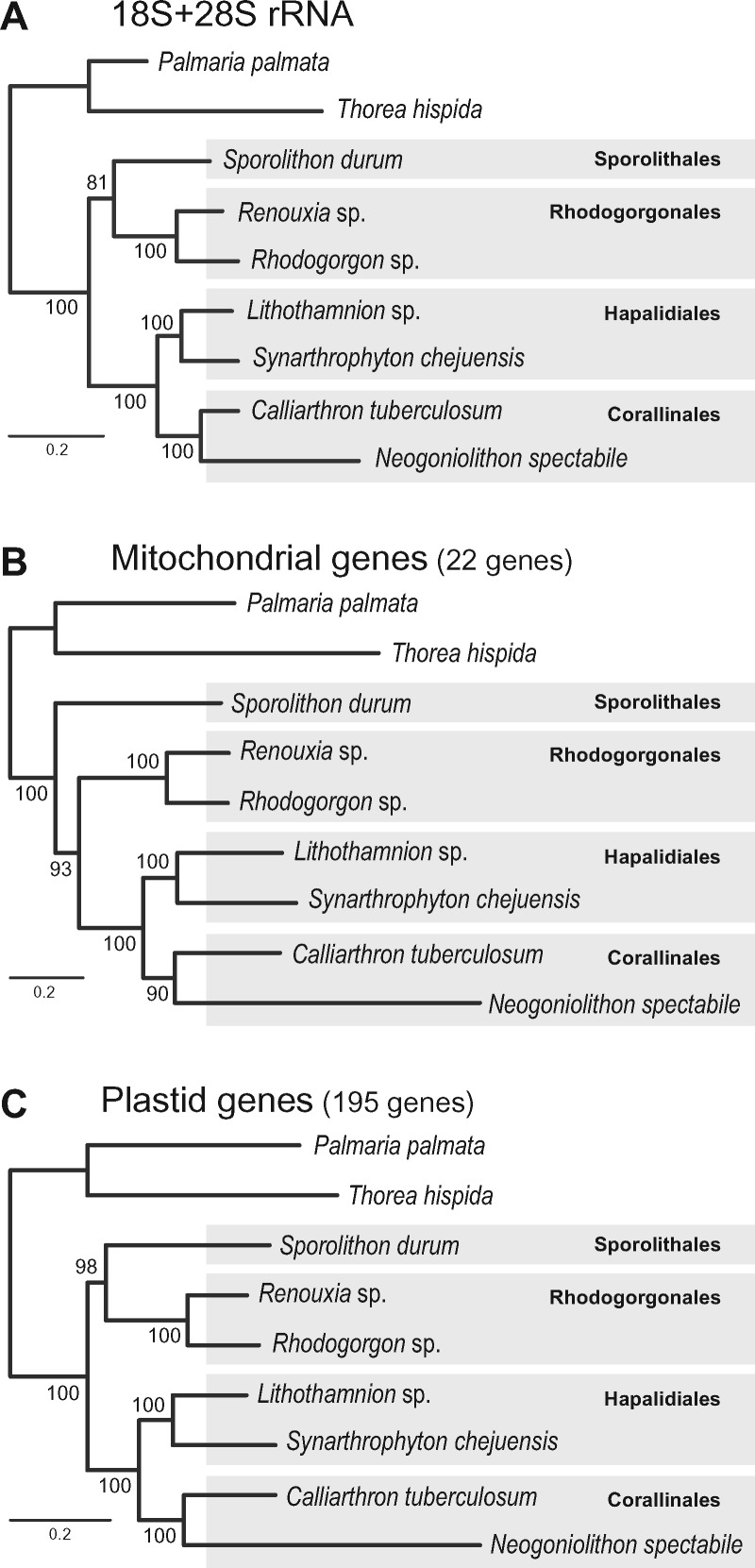
—Maximum likelihood (ML) trees using nuclear ribosomal RNAs (rRNAs) and organelle genes from six Corallinophycidae and two Nemaliophycidae (outgroup) species. (*A*) ML tree built using concatenated 18S and 28S rRNAs. (*B*) ML tree built using aligned 22 concatenated proteins from mitochondrial genomes. (*C*) ML tree built using aligned 195 concatenated proteins from plastid genomes.

It has been generally accepted that the order Sporolithales is the earliest branching group of coralline red algae, based on the application of different molecular markers (Le Gall and Saunders 2007; Nelson et al. 2015), as well as the fossil record, although fossils of Rhodogorgonales species are unknown (Aguirre et al. 2000; 2010). To study the genealogical history of coralline algae, we compared four ML trees using the complete sequences of concatenated rRNAs (18S + 28S rRNA), mitochondrial (cMT; 22 mitochondrial genes) and plastid genes (cPT; 195 plastid genes) (fig. 1). The ML tree of concatenated rRNAs showed two monophyletic clusters (fig. 1*A*), one comprised of Sporolithales (*Spo.durum*) and Rhodogorgonales (*Renouxia* sp. and *Rhodogorogon* sp.) with moderate support (BS: 81%), and the other of Hapalidiales (*Lithothamnion* sp. and *S. chejuensis*) and Corallinales (*C. tuberculosum* and *N.spectabile*) with strong support (BS: 100%). To compare the genealogical histories of these coralline nuclear rRNAs, we constructed ML trees of each rRNA data set, and recovered two different tree topologies (supplementary fig. S7, Supplementary Material online). The ML tree of 18S rRNA showed that the early divergence of Sporolithales was followed by the Rhodogorgonales, with a monophyletic cluster formed by Hapalidiales and Corallinales. It is worth noting that the bootstrap supporting (BS) value for the divergence point of the Rhodogorgonales was relatively low (BS: 51%; supplementary fig. S7*A*, Supplementary Material online). In contrast, the ML tree of 28S rRNA (i.e., a member of the same operon) showed an identical topology to the concatenated rRNA tree (i.e., the monophyly of the Sporolithales and Rhodogorgonales with moderate support, BS: 83%; supplementary fig. S7*B*, Supplementary Material online). Interestingly, these two classes of conflicting topologies were recovered when we used mitochondrial (fig. 1*B*) and plastid (fig. 1*C*) genome data (all branches BS ≥ 90%).

To address this conflict, we analyzed individual gene data sets from each organelle genome (mtDNA 22 and ptDNA 195 genes, respectively). We determined which individual gene supports (or rejects) these two alternative topologies being addressed using the AU test. One mitochondrial and 18 plastid gene data significantly rejected the mitochondrial tree topology (*P*-value ≤ 0.05; supplementary table S4, Supplementary Material online), suggesting that 8.7% of organelle genes have strong conflicts with regard to the basal position of *Spo.durum* (i.e., mitochondrial tree topology).

### Analysis of Individual Gene Phylogenies in Coralline Organelle Genomes

**Fig. 2.**
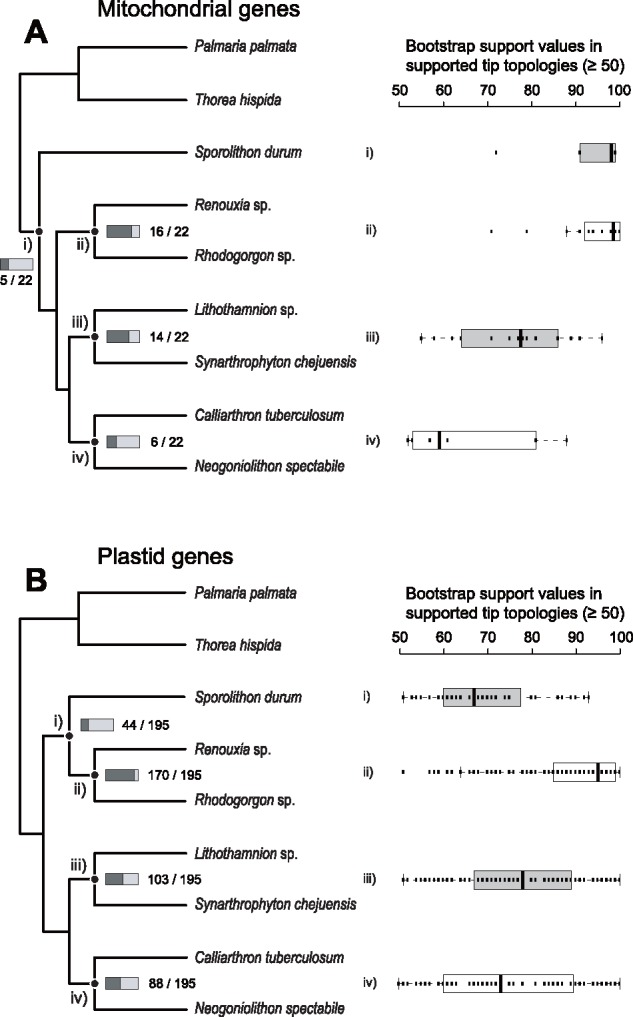
—Numbers of supported tip topologies and their bootstrap support values (≥ 50%) in the concatenated mitochondrial and plastid gene phylogeny each using ML trees of organelle genes. (*A*) Supported tip topologies of mitochondrial gene phylogeny. (*B*) Supported tip topologies of plastid gene phylogeny. The proportions and numbers around the rectangles at the species divergence points (*i*–*iv*) in trees indicate the number of supporting genes at that node. Bootstrap support values are plotted on the right side of the panel.

To identify the phylogenetic signal that underlies the conflict in tree topologies, we compared the terminal branching patterns from all ML trees of individual mitochondrial and plastid genes to the concatenated mtDNA and ptDNA (cMT and cPT) topologies. We counted all observed gene numbers that show the monophyly (BS ≥ 50%) of target species to other species or to the group of species at each divergence point (supplementary figs. S8 and S9, Supplementary Material online). For example, the five genes *nad*5 (BS 99%), *rps*3 (BS 99%), *nad*4 (BS 98%), *rpl*16 (BS 91%), and *nad*1 (BS 72%) all showed a monophyletic relationship between *Spo. durum* and the other six coralline species (fig. 2*A*-*i*). Additionally, the *atp*8 gene phylogeny supported this topology (BS 86%), but the *atp*8 gene of *N.spectabile* was absent due to sequence degradation (low similarity of 428 bp of intergenic sequences between two conserved flanking genes) in the mtDNA (supplementary fig. S8*A*, Supplementary Material online). A total of six mitochondrial gene phylogenies (*atp*8, *nad*5, *rps*3, *nad*4, *rpl*16, and *nad*1) supported the early divergence of the Sporolithales (i.e., *Spo. durum*; supplementary fig. S8*A*, Supplementary Material online) with strong support (i.e., BS = 72–99%). However, the *atp*8 and *nad*1 ML trees showed the same tree topology as the cMT phylogeny (BS values in all branches ≥ 50%). The monophyletic cluster of Sporolithales and two Rhodogorgonales species was supported by five mitochondrial genes (*ymf*39, *cox*1, *cox*3, *nad*2, and *cob*; supplementary fig. S8*A*, Supplementary Material online), but only the *nad*2 gene showed the same tree topology to the cPT phylogeny (BS in all branches ≥ 50%). Although there were small numbers of identical topology patterns within the cMT or cPT phylogeny, we postulate that the mitochondrial genes of these seven corallines contained both classes of genetic information that support the conflicting topologies: that is, the early divergence of Sporolithales (e.g., cMT topology), and the early divergence of the clades Sporolithales + Rhodogorgonales and Corallinales + Hapalidiales (e.g., cPT topology).

The monophyly of Rhodogorgonales and of Hapalidiales was supported by 16 and 14 mitochondrial genes with high BS values, respectively (fig. 2*A*-*ii* and *iii*, and supplementary fig. S8*B*–*E*, Supplementary Material online). However, the monophyletic cluster of Corallinales species (*C. tuberculosum* and *N.spectabile*) was found in only six mitochondrial gene phylogenies with relatively low BS values (fig. 2*A*-*iv*, supplementary fig. S8*F*–*H*, Supplementary Material online). The unstable phylogenetic behavior of Corallinales was primarily caused by *N.spectabile* because, excluding the six mitochondrial genes, the phylogenetic position of this species was not consistent across genes (supplementary fig. S8*G*, Supplementary Material online). In contrast, *C. tuberculosum* clustered with *Neogoniolithon* (six genes) or was sister to the Hapalidiales species (five genes; supplementary fig. S8*F*, Supplementary Material online).

On the basis of 195 ML trees using individual plastid genes, the cluster of *Spo. durum*—Rhodogorgonales (i.e., the cPT phylogeny) was recovered from 44 plastid genes among 134 reliable topologies (BS ≥ 50% in any *Spo. durum* clades), but these relevant BS values were relatively low (average BS: 69.2%; fig. 2*B*-*i* and supplementary fig. S9*A*, Supplementary Material online). Among these 44 plastid genes, however, only 18 plastid genes supported the cPT topology (BS in all branches ≥ 50%; supplementary table S5, Supplementary Material online), which contains the cluster *Spo. durum*—Rhodogorgonales and the other corallines (pattern 1 in supplementary fig. S9*A*, Supplementary Material online). Interestingly, two other topology patterns were present in the plastid gene phylogenies of *Spo. durum* (patterns 2 and 3 in supplementary fig. S9*A*, Supplementary Material online). One was the early divergence of the Sporolithales as shown in the cMT topology that was supported by 44 plastid genes; however, if different subtopology patterns were excluded, only five plastid gene phylogenies were identical to the cMT phylogeny (BS in all branches ≥ 50%; supplementary table S5, Supplementary Material online). The other was the monophyly of *Spo. durum* and the Hapalidiales-Corallinales clade that was supported by 27 plastid genes. Among these genes, one additional tree pattern encompassing 10 plastid gene phylogenies supported the early divergence of Rhodogorgonales, followed by the divergence of *Spo. durum*, and then by the monophyly of Hapalidiales and of Corallinales (BS in all branches ≥ 50%; supplementary table S5, Supplementary Material online).

The monophyletic orders of the Rhodogorgonales (170 genes; fig. 2*B*-*ii*), Hapalidiales (103 genes; fig. 2*B*-*iii*) and Corallinales (88 genes; fig. 2*B*-*iv*) were well-supported, among 182 plastid genes (fig. 2*B* and supplementary fig. S9*H*, Supplementary Material online), although there were frequent incongruent topology patterns in the Corallinales (*Calliarthron* and *Neogoniolithon*) that was found using mitochondrial genes (supplementary fig. S9*B*–*H*, Supplementary Material online). These incongruences were already reported from previous studies including a larger coralline taxon sample using one nuclear and three plastid markers (18S rRNA, *psa*A, *psb*A and *rbc*L; Nelson et al. 2015). In summary, we found three major phylogenetic tree topology patterns among coralline species using individual gene analysis.

In addition, we analyzed subsampled data set-based phylogenies of mitochondria and plastid genes using the TIGER program, that progressively excludes highly variable sites from the alignments (Cummins and McInerney 2011). We constructed subsampled data sets from concatenated mitochondria and plastid alignments with high-level gradient (option: -b 50), and then generated the phylogeny using IQ-tree. The proportions of maximum subsampled data set were 53% (plastid) and 60% (mitochondria) of alignments, which indicates ∼40% of highly variable amino acid sites (supplementary fig. S10, Supplementary Material online). When we excluded these highly variable sites, the tree topologies were unchanged both in mitochondrial and plastid gene analyses, however support values gradually decreased only in node “b” (i.e., monophyly of all coralline species except *Spo.durum*; supplementary fig. S10*A*, Supplementary Material online). Similar trends were found in the plastid data sets. Tree topologies were not changed until 38% of the original data set was used. Bootstrap support of node “b” (i.e., monophyly of *Spo. durum* and two Rhodogorgonales species) gradually decreased (see supplementary fig. S10*B*, Supplementary Material online). When we used this subsampling strategy on individual genes, most nodes collapsed with very short branches, likely caused by insufficient phylogenetic signal (results not shown). Thus, we found that mitochondrial and plastid tree topologies are well conserved across most nodes when using conserved or variable sequences. However, two particular nodes (see above) are supported only by highly variable sequences.

### Three Evolutionary Scenarios of Coralline Organelle Genomes

**Fig. 3.**
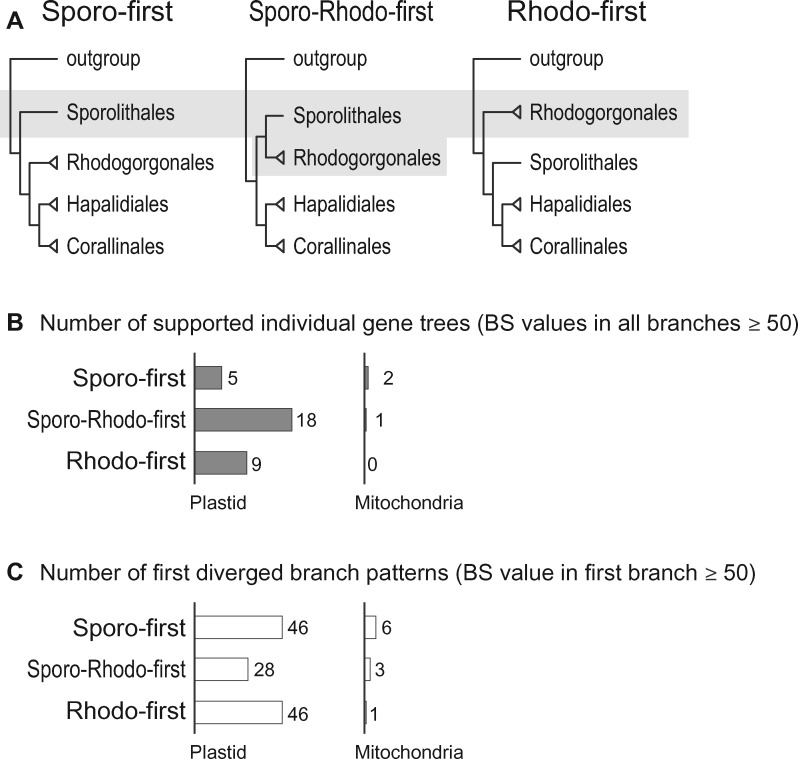
—Major topologies of individual organelle gene phylogenies among coralline species. (*A*) The three major topology categories reflecting the early diverging coralline order(s). (*B*) Number of organelle gene phylogenies supporting each topology category (BS values in all branches ≥ 50%). (*C*) Number of early diverging patterns (BS values in first branch ≥ 50%). Abbreviations: Sporo-first and S = Sporolithales-first scenario, Sporo-Rhodo-first and SR = Sporolithales–Rhodogorgonales-first scenario, Rhodo-first and R = Rhodogorgonales-first scenario.

On the basis of these tree topologies, we propose three alternate evolutionary scenarios to summarize coralline organelle genome evolution: 1) the Sporolithales diverged first (Sporo-first), 2) the cluster Sporolithales–Rhodogorgonales diverged first (Sporo-Rhodo-first), and 3) the Rhodogorgonales diverged first (Rhodo-first; fig. 3*A*). These scenarios are reflected in the fossil record in which the Sporolithales diverged earlier than the Hapalidiales and Corallinales (Aguirre et al. 2000, 2010), although fossils of Rhodogorgonales have not yet been found because thalli in this lineage are less extensively calcified. Although these three evolutionary scenarios were supported by only circa 10% of genes from two organelle genome data (mtDNA: three genes and ptDNA: 32 genes; fig. 3*B*), we suggest that these complex evolutionary histories could be explained by ILS, in particular among the ancestors of Sporolithales and Rhodogorgonales. By this we mean that individual gene trees conflict with the overall genome tree because some alleles, surprisingly, failed to coalesce during the several hundred million years of coralline algal evolution. To identify potential ILS-impacted organelle genes, we focused on the early diverged taxa when compared with the outgroup (BS value in first branch ≥ 50%; fig. 3*C* and supplementary table S6, Supplementary Material online) regardless of other internal relationships (i.e., ignoring potential independent gene mutations in ingroup taxa). Almost one-half of each organelle genome (mtDNA: 10 genes and ptDNA: 120 genes) is involved in the conflicting phylogenetic signal in the early diverging branches, particularly in the plastid genome data (fig. 3*C*). To determine whether natural selection played a (dominant) role in the spectrum of genes putatively impacted by ILS, we compared the functional categories of these genes that support different topologies. This analysis provided no obvious evidence of correlation between gene function and the three evolutionary scenarios (Sporo-Rhodo-first/Sporo-first/Rhodo-first; supplementary table S6, Supplementary Material online) including ribosomal proteins (5/9/11 genes), photosystem related proteins (0/7/3 genes) and cytochrome related proteins (1/4/2 genes). In addition, each gene category was not highly clustered or concentrated in a specific region of organelle genomes (supplementary fig. S11, Supplementary Material online). On the basis of these comparisons, it is likely that there was no selection acting on the retention of ancestral polymorphisms with regard to gene function or genome structure in the different evolutionary histories of coralline organelle genomes (see test of diversifying selection below).

**Fig. 4.**
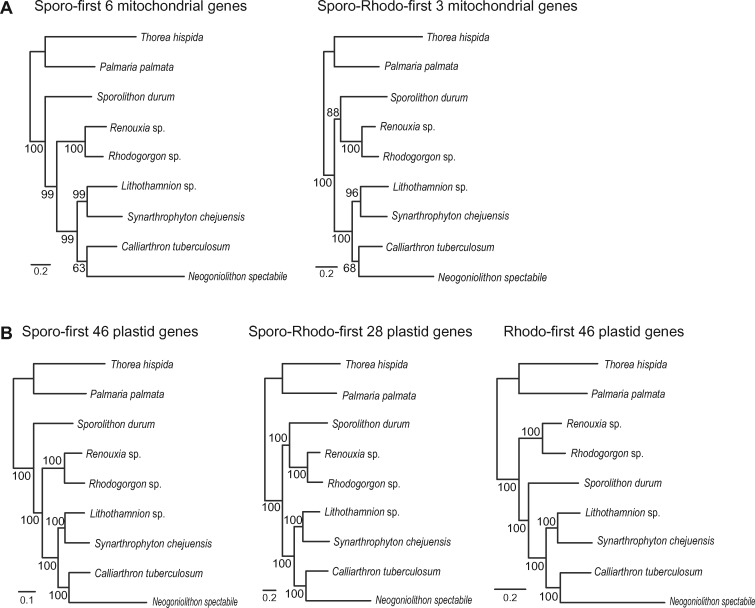
—Concatenated ML trees built using the mitochondrial and plastid genes that support the major evolutionary scenarios. (*A*) ML trees of two competing topologies built using concatenated mitochondrial genes. (*B*) ML trees of three conflicting topologies built using concatenated plastid genes.

To compare phylogenetic relationships of coralline species under the three different evolutionary scenarios, we constructed concatenated ML trees using each differently categorized organelle genes: six mitochondrial genes for Sporo-first, three mitochondrial genes for Sporo-Rhodo-first (fig. 4*A*), 46 plastid genes for Sporo-first, 28 plastid genes for Sporo-Rhodo-first, and 46 plastid genes for Rhodo-first scenarios (fig. 4*B*). As expected, these different evolutionary scenarios were supported with high BS values (most of BS values = 96–100%; fig. 4), although the monophyletic relationship of Corallinales species shows relatively low BS values in mitochondrial phylogenies (63–68%; fig. 4*A*), likely due to the long branches of *N.spectabile*. The monophyletic relationship of Hapalidiales and Corallinales was recovered in our study consistent with the fossil record (Aguirre et al. 2000, 2010). However, Sporolithales and Rhodogorgonales contained at least 2–3 different evolutionary histories in their organelle genomes.

To test whether these genes had undergone different selective pressures with respect to sequence evolution under the different evolutionary scenarios, we analyzed using the PAML package (alignment-based pairwise comparisons; runmode = -2; Yang 2007) nonsynonymous substitution rates (dN) of these genes between coralline species and the outgroup *Palmaria palmata* (supplementary table S7 and fig. S12, Supplementary Material online). There were, however, similar patterns of nonsynonymous substitution between coralline species whether mitochondria (supplementary fig. S12*A*, Supplementary Material online) or plastid genes (supplementary fig. S12*B*, Supplementary Material online) were investigated. In addition, there were no significant differences between Sporolithales and Rhodogorgonales species (*P*-value > 0.1; Wilcoxon rank sum test; supplementary table S8, Supplementary Material online). On the basis of these results, it is hard to determine if these conflicts are derived from atypical mutation patterns in some organellar genes or in particular species. Therefore, we postulate that the incongruent topologies between the Sporolithales and Rhodogorgonales (e.g., fig. 1*B* and *C*) could be explained by ILS in the stem lineages of corallines resulting from a rapid radiation (i.e., fig. 3*C*) of taxa that contained many ancestral polymorphisms (supplementary fig. S10, Supplementary Material online). The rapid radiation of Florideophyceae, including the Corallinophycidae has already been reported (Lee, Cho, et al. 2016). In angiosperms, chloroplast phylogenomic analysis of 53 grape species shows incongruent phylogenetic relationships that were explained by both hybridizations and a rapid radiation (Wen et al. 2018). Phylogenomic analysis of the sunflower *Espeletia* using a large taxon sampling (41 species) also showed explosive adaptive radiation-derived ILS (Pouchon et al. 2018). A similar case revealed that the nuclear and mitochondrial phylogenies showed conflicts due to the ILS and introgression within the bear lineage (Kutschera et al. 2014; Kumar et al. 2017). A genome-wide analysis uncovered ILS in modern birds caused by a rapid radiation, particularly among early-diverged species (Jarvis et al. 2014). Another possible explanation is that the complex evolutionary history of coralline organelle genomes reflects gene flow by natural hybridization between ancestral coralline species.

Because such phylogenetic incongruence was also found in two nuclear rRNA markers (i.e., 18S and 28S rRNAs; fig. 1*A* and *B*), we presume that the coralline nuclear genomes may also contain a complex evolutionary history. Complex hybridization or ILS, as well as a high level of divergence (homoplasy, i.e., highly variable sequences) could explain these incongruences. For instance, potential hybridization has been suggested for the coralline genus *Chiharaea* (Corallinales) based on phylogenetic analyses using the nuclear, mitochondrial, and plastid markers (ITS, COI, *rbc*L, and *psb*A; Hind and Saunders 2013). However, to test the hypothesis of rapid radiation-derived ILS in the coralline algae, additional taxa need to be studied with their nuclear genomes.

**Fig. 5.**
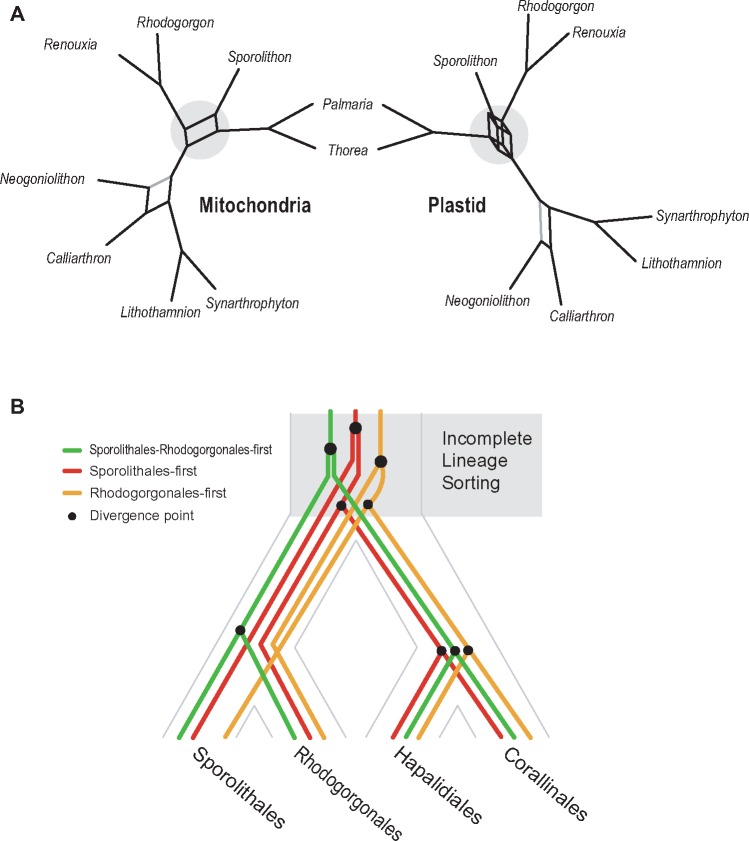
—Incongruent phylogenetic history among coralline organelle genomes. (*A*) Intertwining phylogenetic trees reflecting the major evolutionary histories encoded by mitochondrial and plastid genomes (grey regions indicate phylogenetic uncertainty). (*B*) Phylogenetic divergence scenarios reflecting incomplete lineage sorting based on the three competing evolutionary histories.

To further study ILS in corallines (i.e., beyond incongruent phylogenies) we merged phylogenetic trees into consensus networks using the intertwining phylogenetic tree method (fig. 5*A*; the cutoff value of proportion from present topology patterns = 0.2; Schliep et al. 2017). For this approach, we only used reliable individual gene trees (all branches BS ≥ 50% with all taxa; supplementary table S9, Supplementary Material online) to minimize error from unresolved phylogenetic nodes, missing taxa, and a high divergence level. The intertwining phylogenetic trees show all possible well-supported phylogenetic variations among coralline organelle genomes (grey color in fig. 5*A*). It would be useful to document such complex phylogenetic relationships including some cryptic relationships. Although we cannot clearly establish “what is the first diverged coralline order within the subclass Corallinophycidae?” due to the cryptic relationship between these early diverged coralline orders, we nevertheless postulate that, from the ancestral divergence point of view regarding coralline species, ILS likely contributed to the different phylogenetic patterns (fig. 5*B*) and generated the conflicts between mtDNA and ptDNA trees and perhaps the intergenic features within each organelle.

## Conclusion

This study investigated the major trends in organelle gene phylogeny among coralline species. At least 600 million years-old (Doushantuo formation; Xiao et al. 1998; Xiao et al. 2004; Condon et al. 2005), the Corallinophycidae is a florideophycean lineage without any report of prominent organelle horizontal gene transfers, including replacement of conserved genes. Therefore, organelle genealogical histories are expected to be consistent. However, a consensus evolutionary history between Sporolithales and Rhodogorgonales using mitochondrial and plastid genome data was difficult to find. There were at least two or three different evolutionary histories apparent in the two organelle genomes, likely caused by ILS. On the basis of the analyses of individual gene trees in coralline species, we found several incongruences in terminal nodes (i.e., species-level), and this might be the case when the ancestors of stem groups (i.e., ordinal-level) diverge into different lineages. Because ILS-derived phylogenetic incongruence is generally understood to be derived from recent rapid radiations, natural hybridization, and introgression (Shaw 2002; Fehrer et al. 2007; Toews and Brelsford 2012; Jarvis et al. 2014; Kumar et al. 2017), we postulate that the individual genes of organelle genomes in coralline red algae underwent a complex evolutionary history independently, but in deep time. Regardless of how it happened, our work demonstrates that care must be taken when analyzing phylogenies based on mitochondrial and plastid markers. If complete nuclear genome data from coralline species with a broad taxon sampling were to become available, these may provide a more detailed understanding of the evolutionary patterns revealed in our study. It is also important to determine if ILS (or other sources of phylogenetic conflict) are widespread among other red algae and therefore poses a potentially significant hurdle to the use of organelle genome-based phylogenies in this phylum.

## Supplementary Material

Supplementary Data
