# Supplementary material for: Mitochondrial and Plastid Genomes from Coralline Red Algae Provide Insights into the Incongruent Evolutionary Histories of Organelles
Source: Genome Biol Evol. 2018 Oct 26;10(11):2961–72. doi: 10.1093/gbe/evy222 (PMC6279150; doi:10.1093/gbe/evy222)
Supplement: Supplementary Data [file evy222_supp.zip › Supplementary figures.pdf]

Mitochondrial and plastid genomes from coralline red algae provide insights into the incongruent evolutionary histories of organelles

JunMo Lee[1], Hae Jung Song[1], Seung In Park[1], Yu Min Lee[1], So Young Jeong[2], Tae Oh Cho[2], Ji Hee Kim[3], Han-Gu Choi[3], Chang Geun Choi[4], Wendy A. Nelson[5,6], Suzanne Fredericq[7], Debashish Bhattacharya[8], and Hwan Su Yoon[1]

[1]Department of Biological Sciences, Sungkyunkwan University, Suwon, 16419, Korea
[2]Department of Marine Life Science, Chosun University, Gwangju 61452, Korea
[3]Division of Life Sciences, Korea Polar Research Institute, KOPRI, Incheon 21990, Korea
[4]Department of Ecological Engineering, Pukyong National University, Busan 48513, Korea
[5]National Institute for Water and Atmospheric Research, Wellington 6241, New Zealand
[6]School of Biological Sciences, University of Auckland, Auckland 1142, New Zealand
[7]Biology Department, University of Louisiana at Lafayette, Lafayette, LA 70504-3602, USA
[8]Department of Biochemistry and Microbiology, Rutgers University, New Brunswick, New Jersey 08901, USA

Supplementary figures

Plastid psbA gene

Mitochondrial cox1 gene

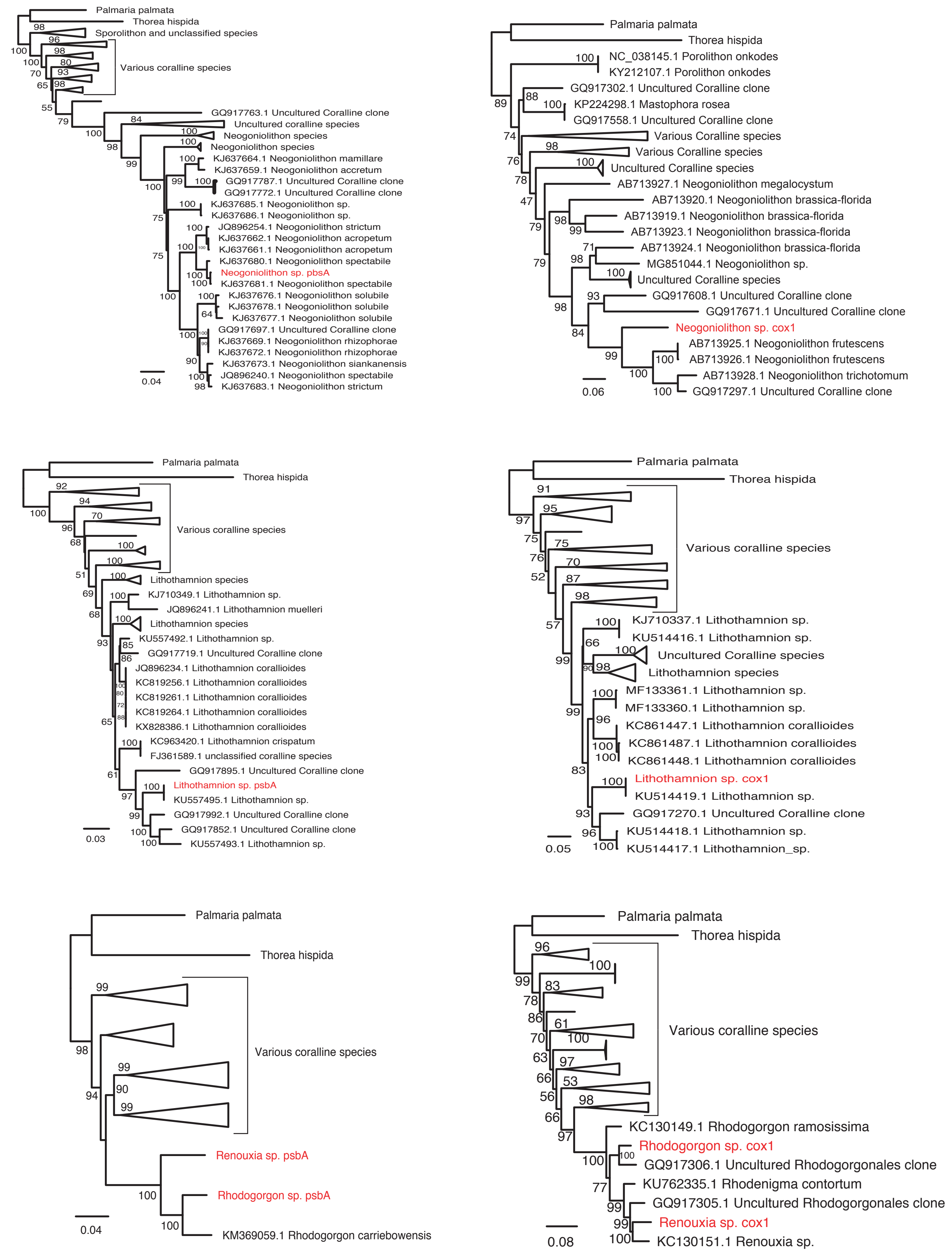

Supplementary fig. S1. Identifications of coralline species using phylogenies of *psb*A and *cox*1 genes (ML trees of aligned nucleotide sequences constructed by IQ-tree program with 1000 replications).

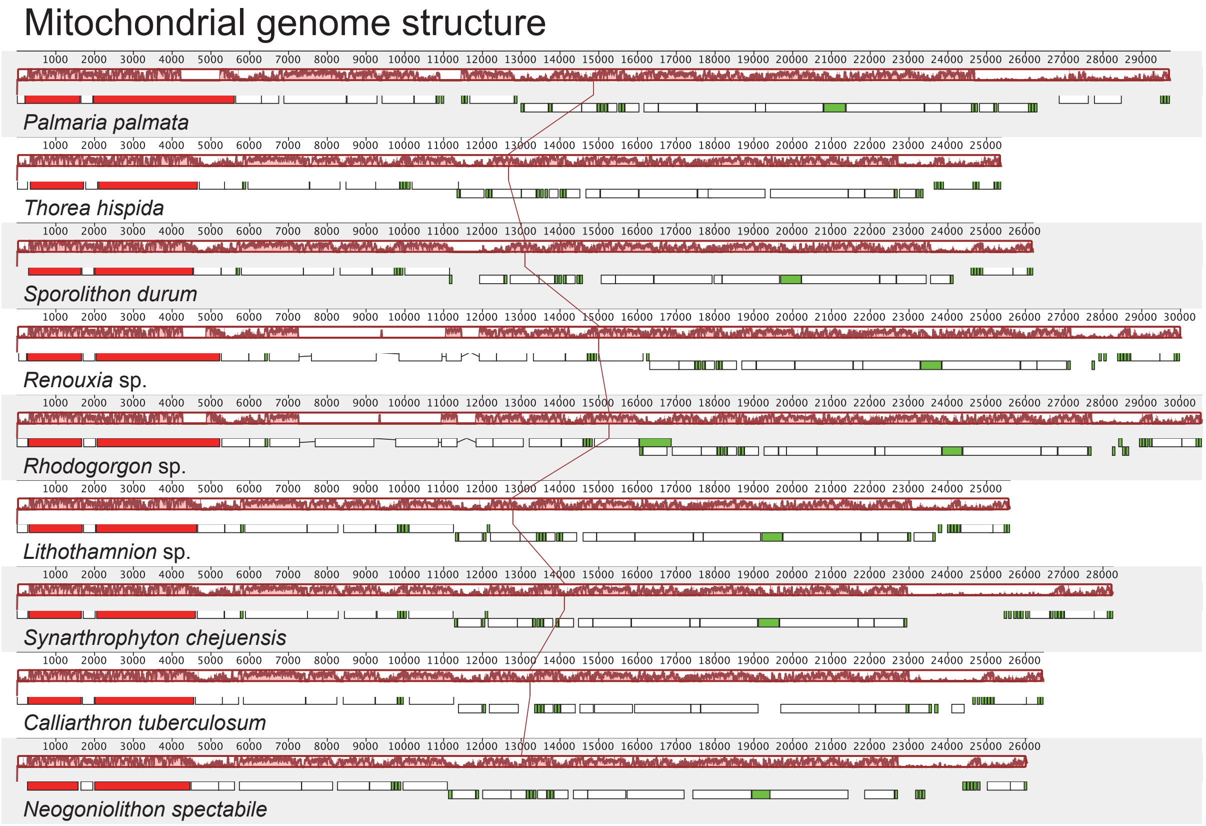

Supplementary fig. S2. Structural comparison of mitochondrial genomes from 6 Corallinophycidae and 2 Nemaliophycidae (outgroup) species based on MAUVE alignment result. The mitochondrial genomes are analyzed by Locally Collinear Blocks (LCB) method in MAUVE. Red boxes on the left edge indicate rRNA operons, whereas synteny blocks with the red lines indicate homologous genome orders based on nucleotide similarity. Simplified gene synteny blocks of each genome are shown under the similarity profiles.

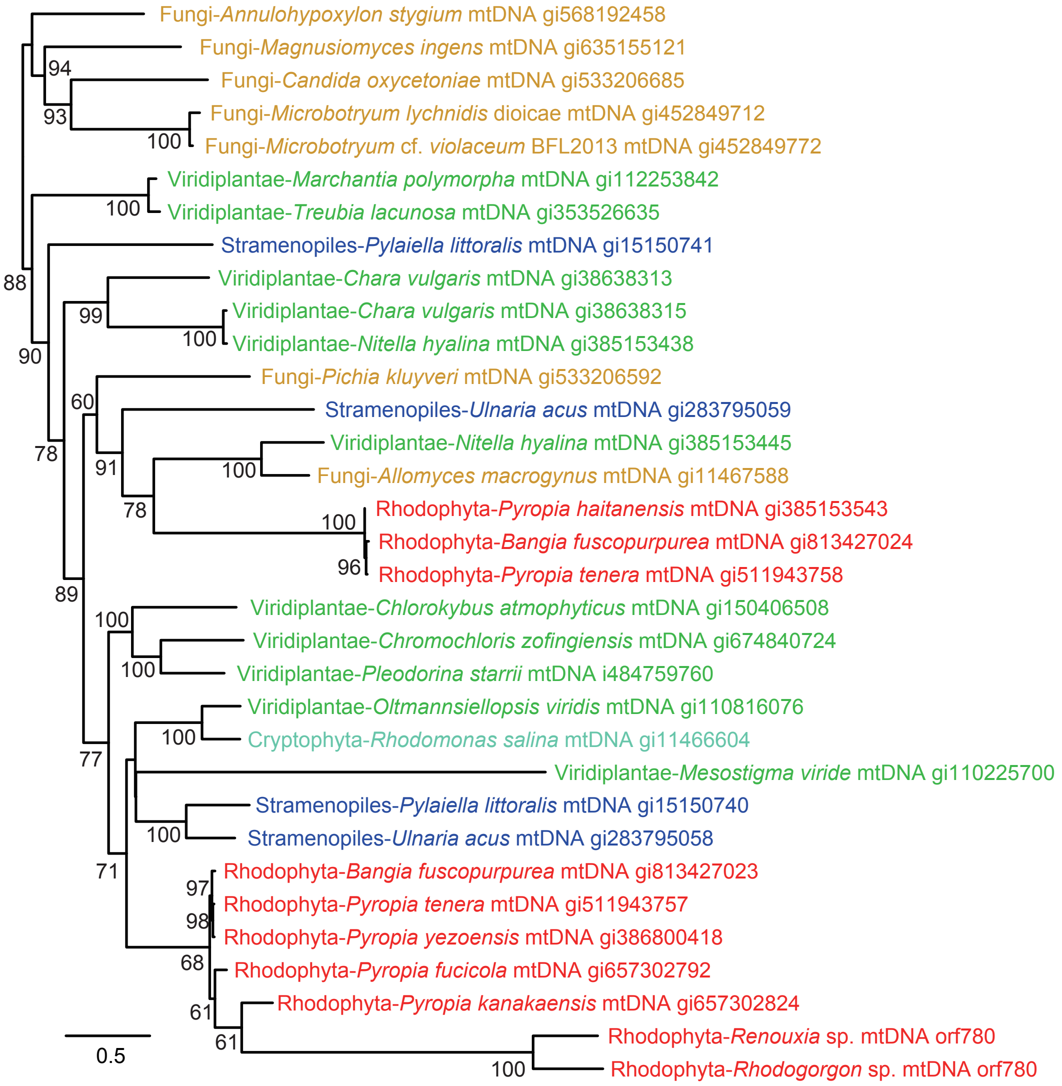

Supplementary fig. S3. ML tree built using aligned homologous genes (Blastp with 1.e-05 e-value cutoff to local RefSeq database) of Rhodogorgonales mitochondrial orf780 (red color: Rhodophyta, blue color: Stramenopiles, green color: Viridiplantae, cyan color: Cryptophyta and light brown color: Fungi).

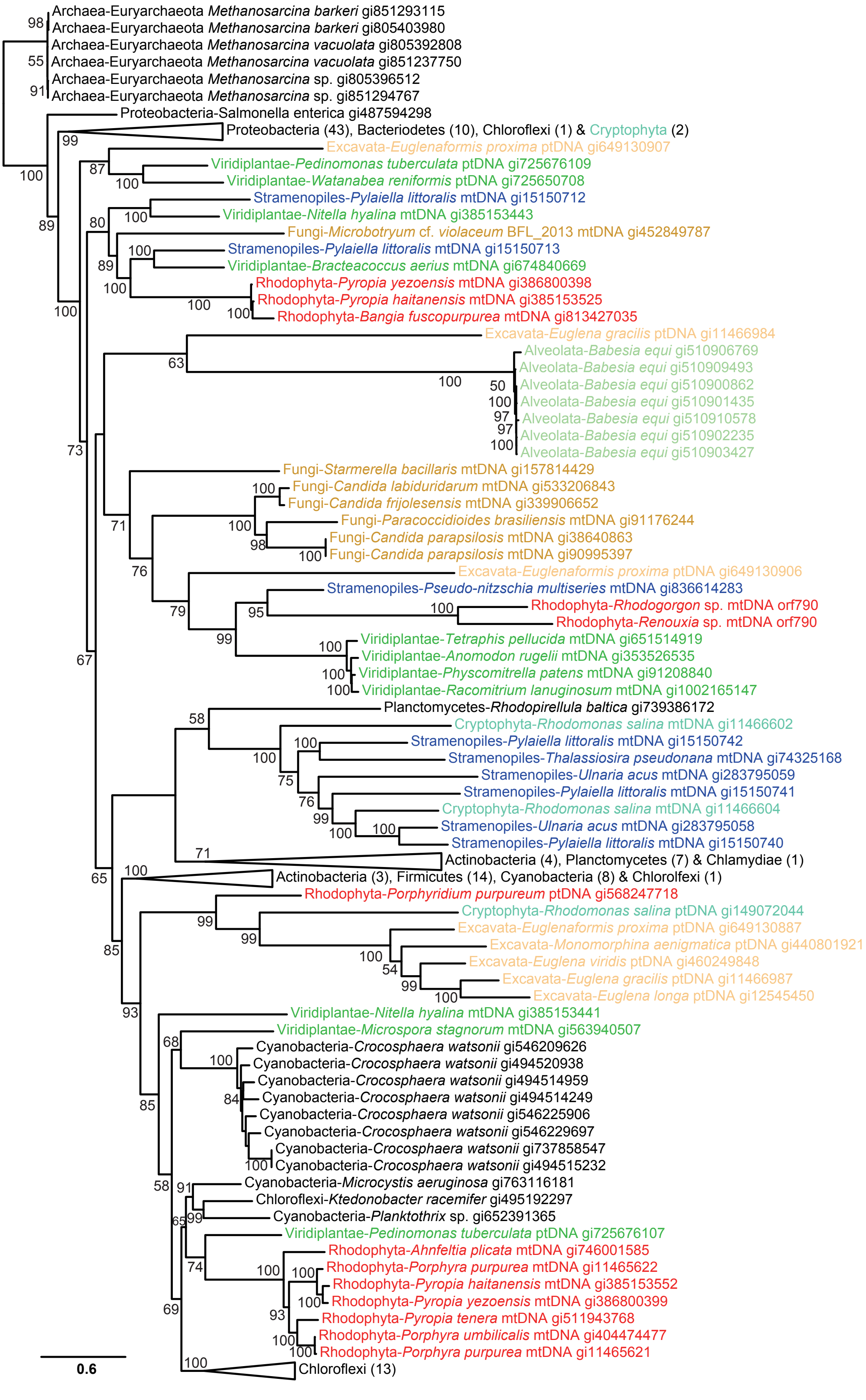

Supplementary fig. S4. ML tree built using aligned homologous genes (Blastp with 1.e-05 e-value cutoff to local RefSeq database) of Rhodogorgonales mitochondrial orf790 (red color: Rhodophyta, blue color: Stramenopiles, green color: Viridiplantae, cyan color: Cryptophyta, light brown color: Fungi, orange color: Excavata and black color: Prokaryotes).

Plastid genome structure

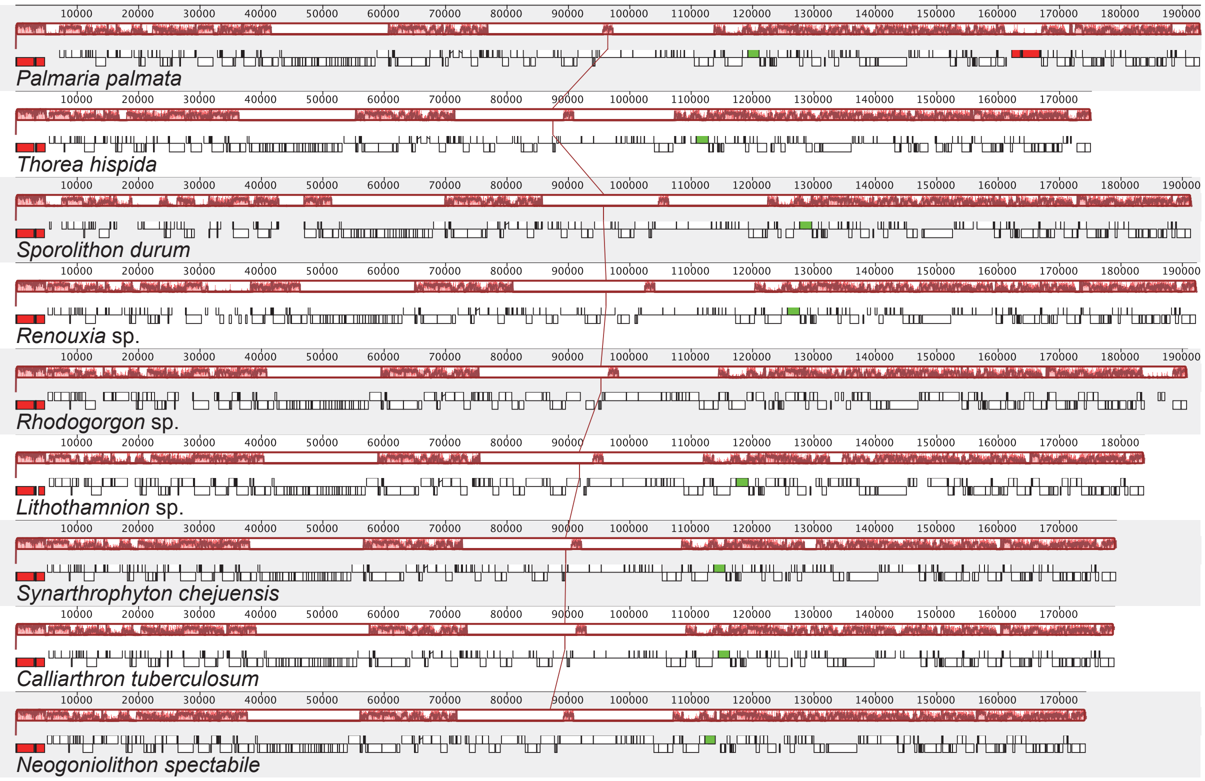

Supplementary fig. S5. Structural comparison of plastid genomes from 6 Corallinophycidae and 2 Nemaliophycidae (outgroup) species based on MAUVE alignment result. The plastid genomes are analyzed by Locally Collinear Blocks (LCB) method in MAUVE. Red boxes on the left edge indicate rRNA operons, whereas synteny blocks with the red lines indicate homologous genome orders based on nucleotides similarity. Simplified gene synteny blocks of each genome are shown under the similarity profiles.

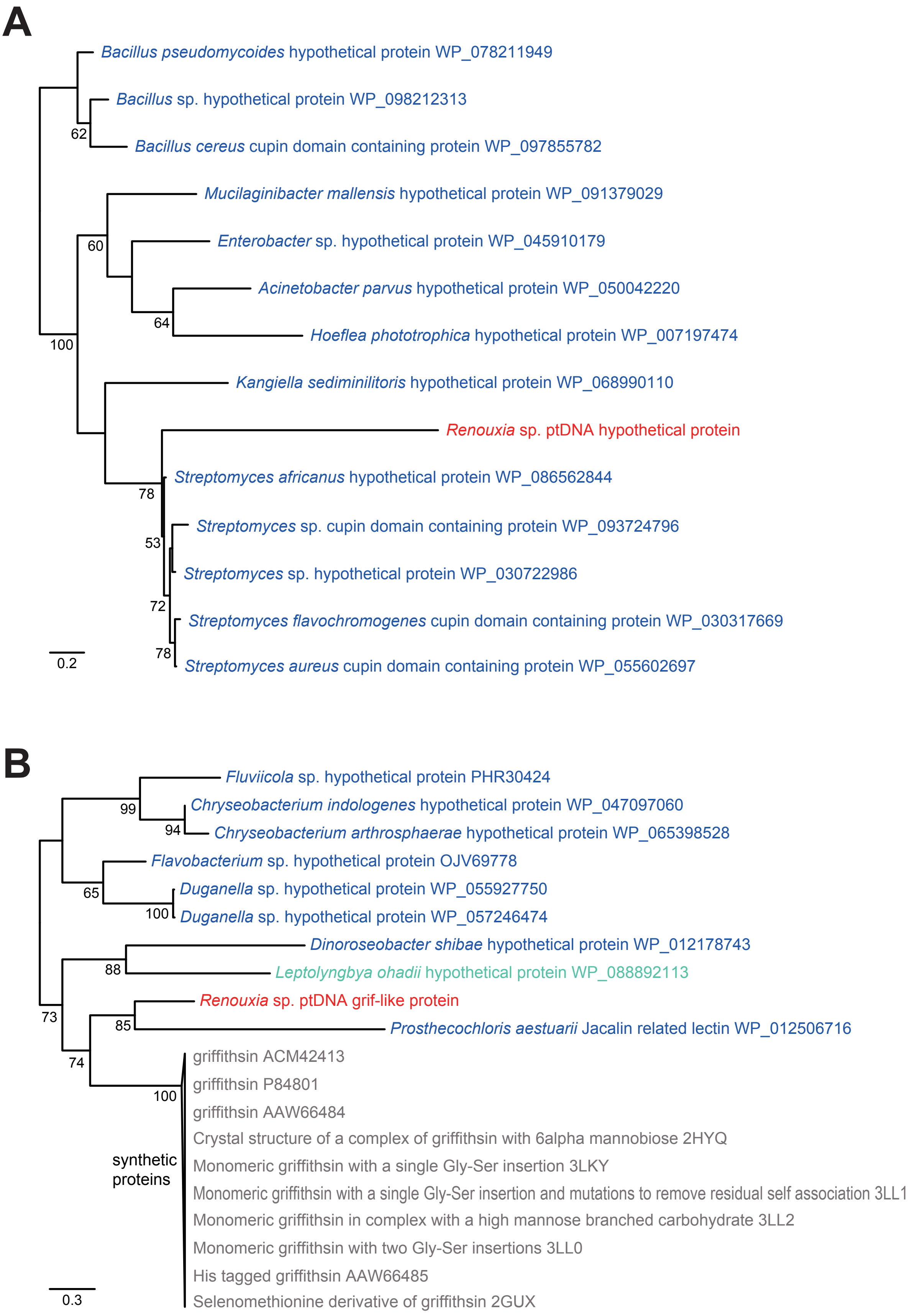

Supplementary fig. S6. Two cases of bacterial horizontal gene transfers in the plasmid-derived sequences of the *Renouxia* plastid genome (red color: hypothetical genes of *Renouxia* sp., blue color: bacteria species, cyan color: cyanobacteria species and grey color: griffithsin-related proteins as synthetic proteins). (*A*) The ML tree built using aligned homologous genes (Blastp with 1.e-05 e-value cutoff to local RefSeq database) of *Renouxia* plastid hypothetical protein. (*B*) The ML tree built using aligned homologous genes (Blastp with 1.e-05 e-value cutoff to local RefSeq database) of *Renouxia* plastid griffithsin-like (grif-like) protein.

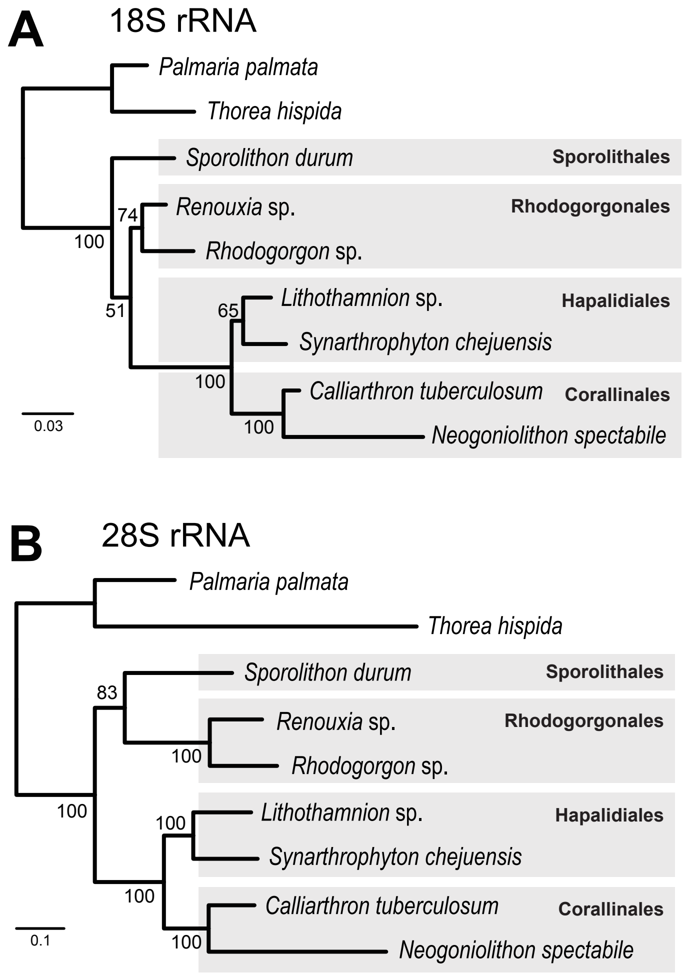

Supplementary fig. S7. Maximum likelihood (ML) trees using nuclear ribosomal RNAs (rRNAs) from 6 Corallinophycidae and 2 Nemaliophycidae (outgroup) species. (A) ML tree built using 18S rRNAs. (B) ML tree built using 28S rRNAs.

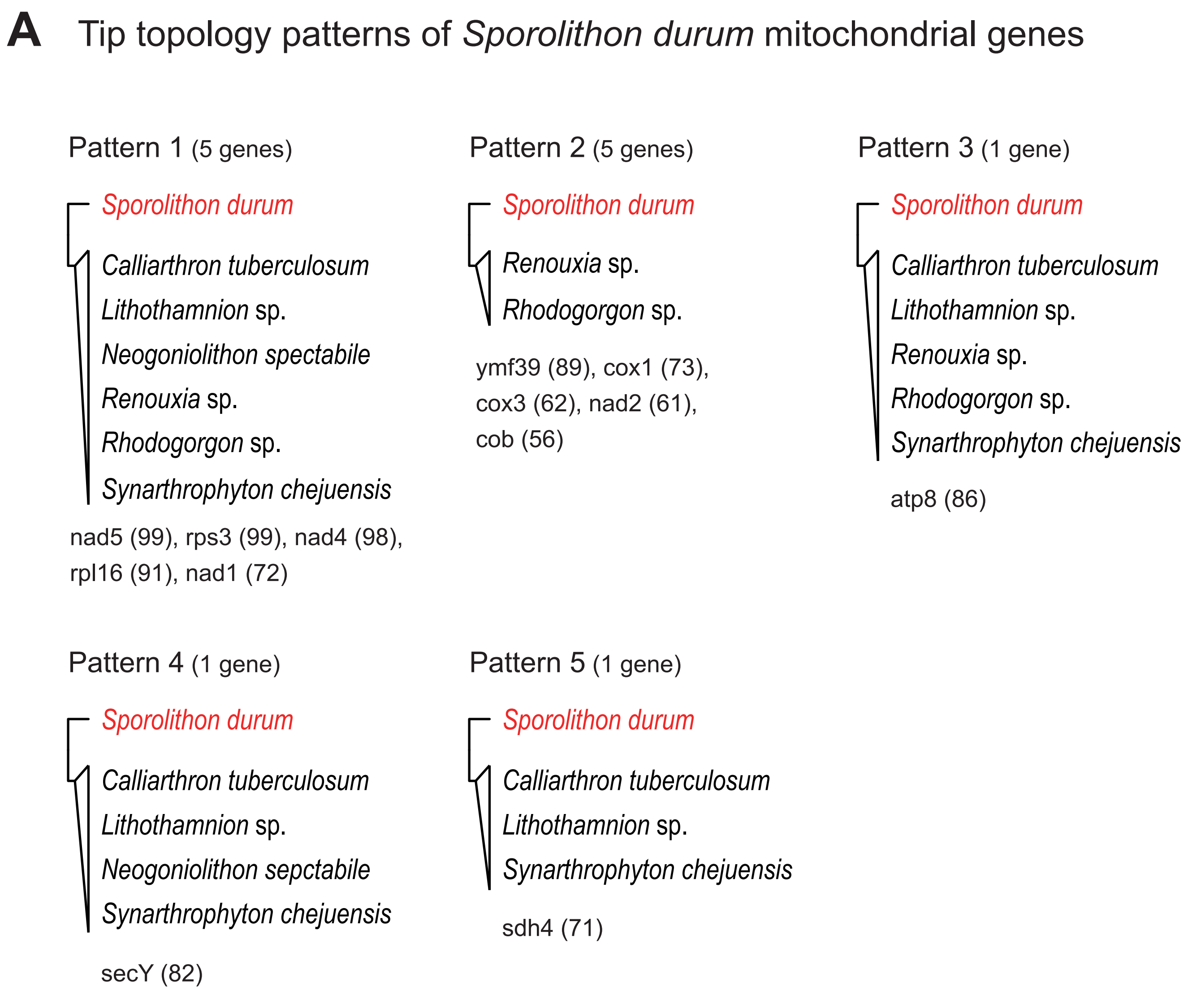

Supplementary fig. S8. The tip topology patterns of mitochondrial gene phylogenies from 7 coralline species. (*A*) Tip topology patterns of *Sporolithon durum*.

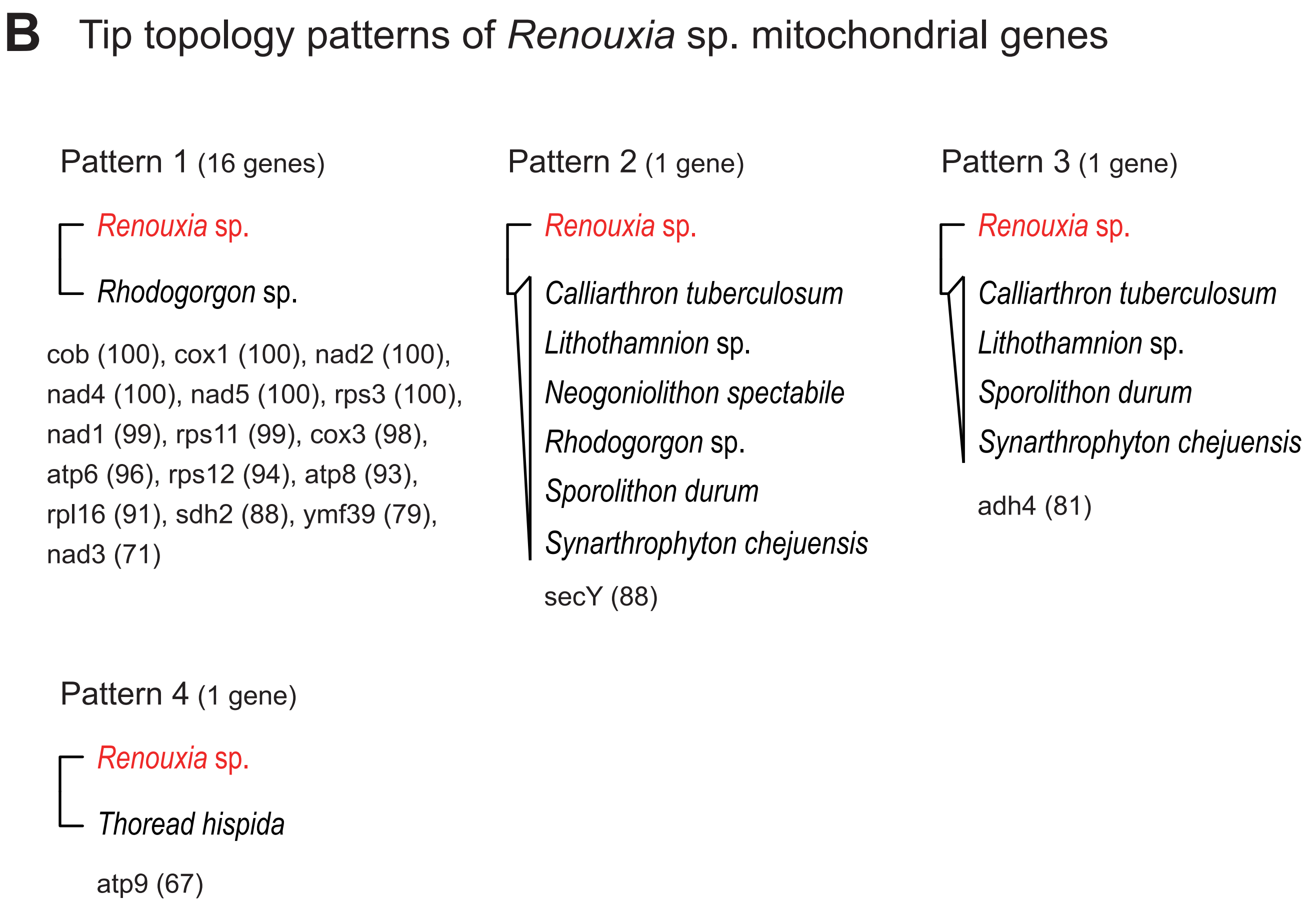

Supplementary fig. S8. The tip topology patterns of mitochondrial gene phylogenies from 7 coralline species. (*B*) Tip topology patterns of *Renouxia* sp.

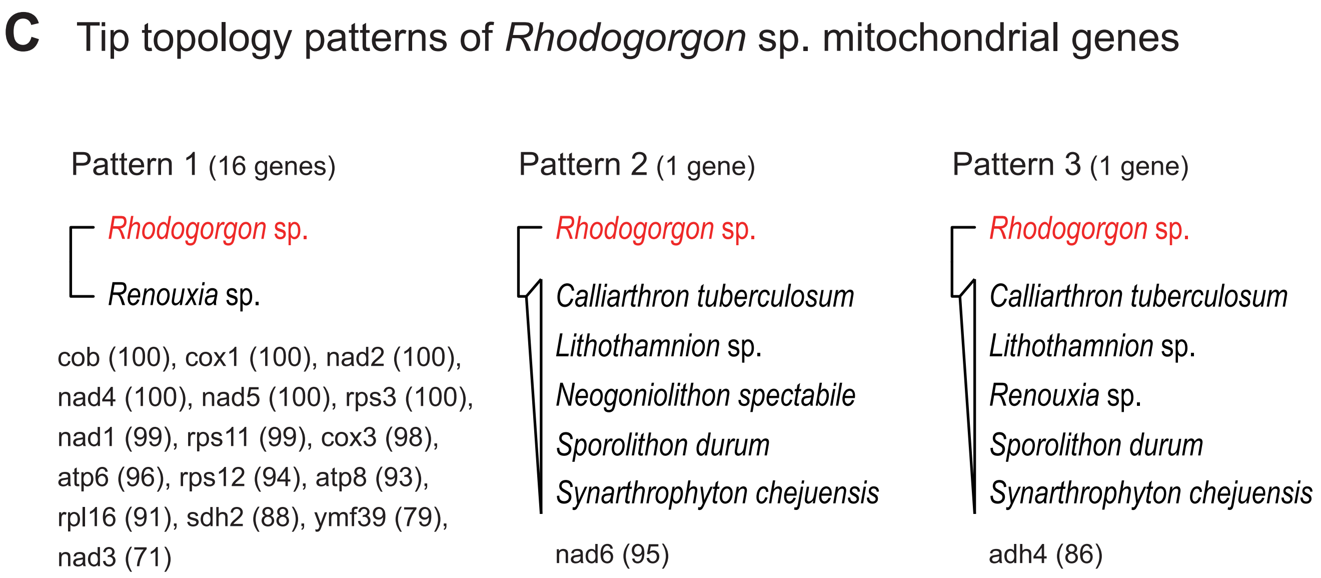

Supplementary fig. S8. The tip topology patterns of mitochondrial gene phylogenies from 7 coralline species. (*C*) Tip topology patterns of *Rhodogorgon* sp.

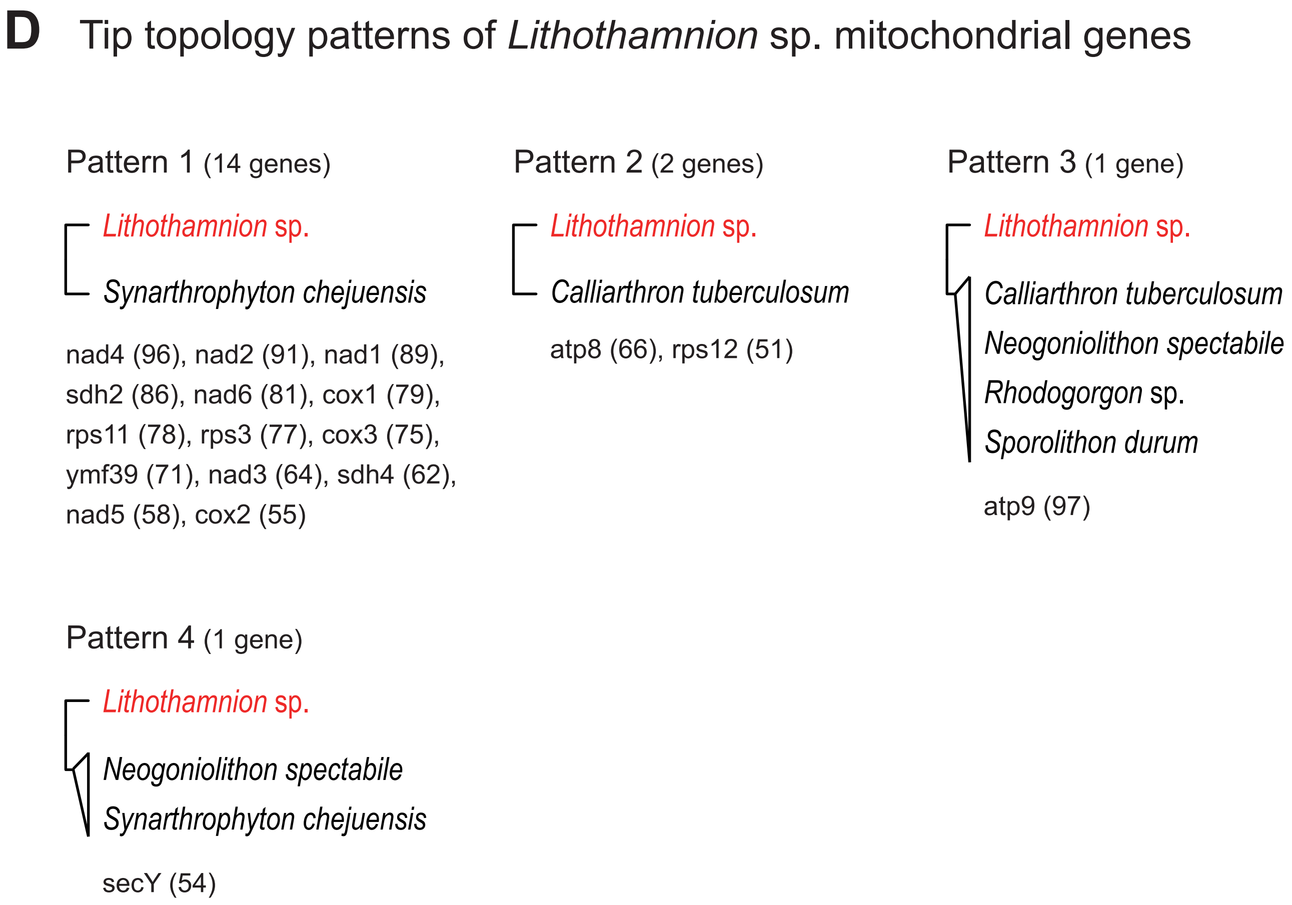

Supplementary fig. S8. The tip topology patterns of mitochondrial gene phylogenies from 7 coralline species. (*D*) Tip topology patterns of *Lithothamnion* sp.

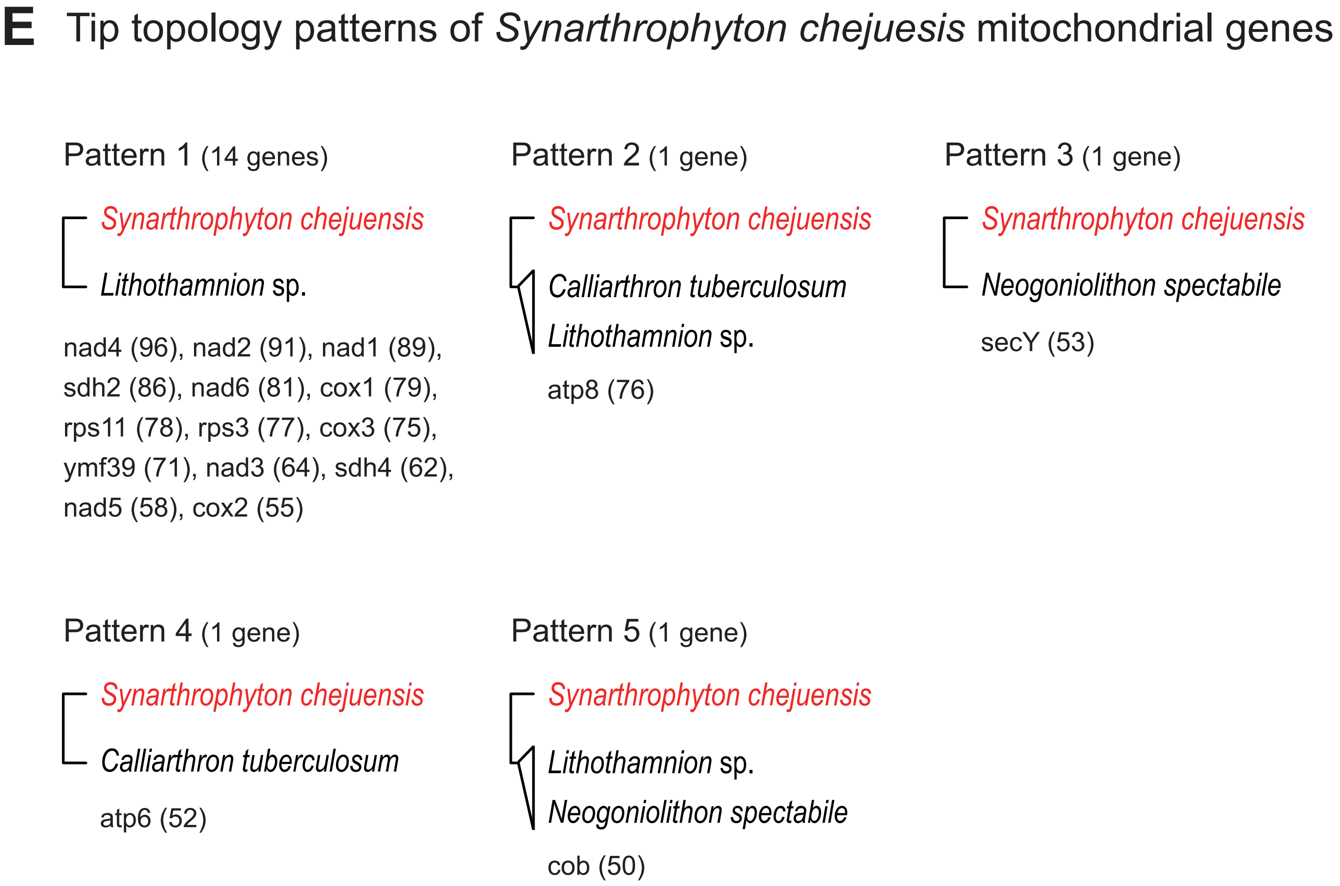

Supplementary fig. S8. The tip topology patterns of mitochondrial gene phylogenies from 7 coralline species. (*E*) Tip topology patterns of *Synarthrophyton chejuensis*.

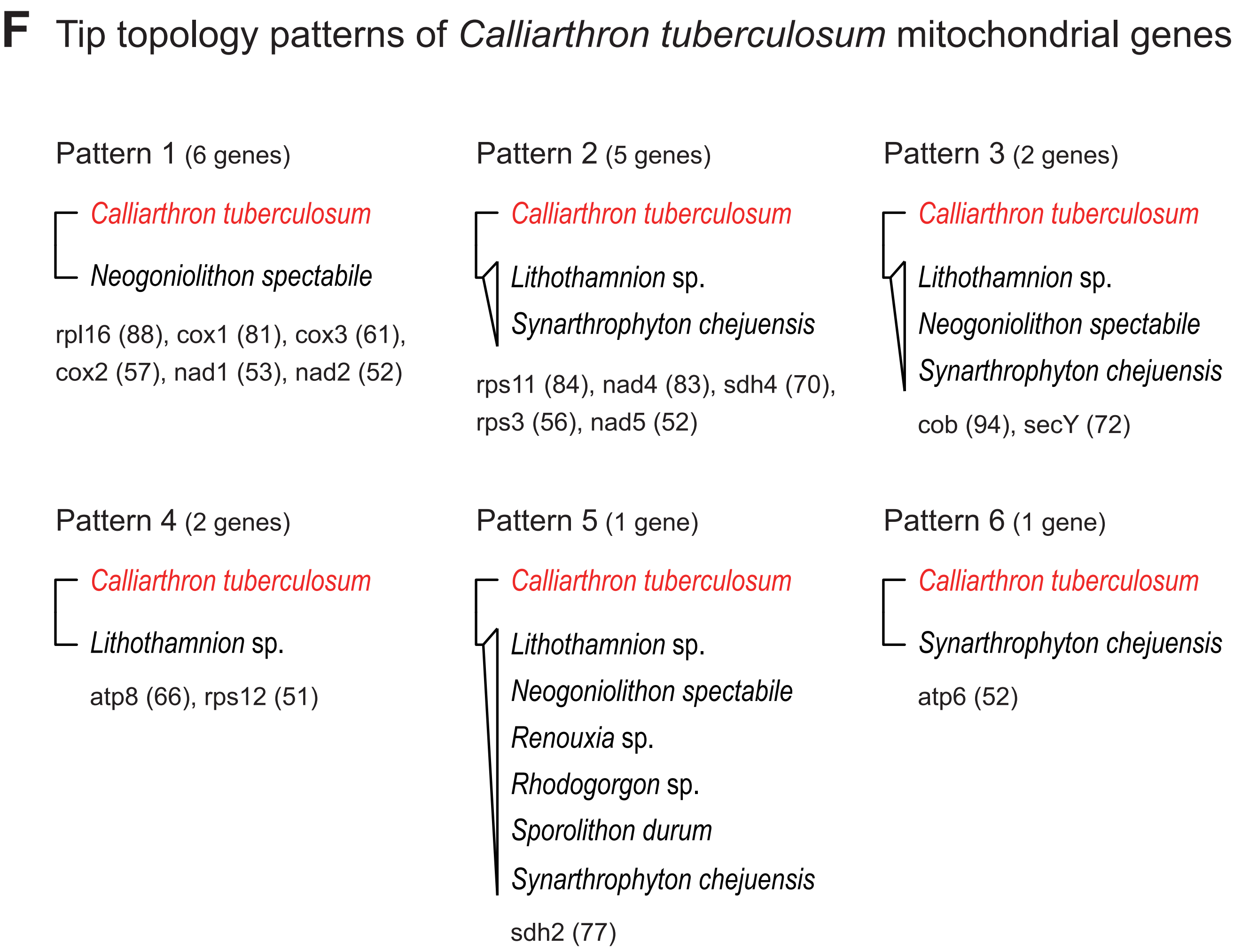

Supplementary fig. S8. The tip topology patterns of mitochondrial gene phylogenies from 7 coralline species. (*F*) Tip topology patterns of *Calliarthron tuberculosum*.

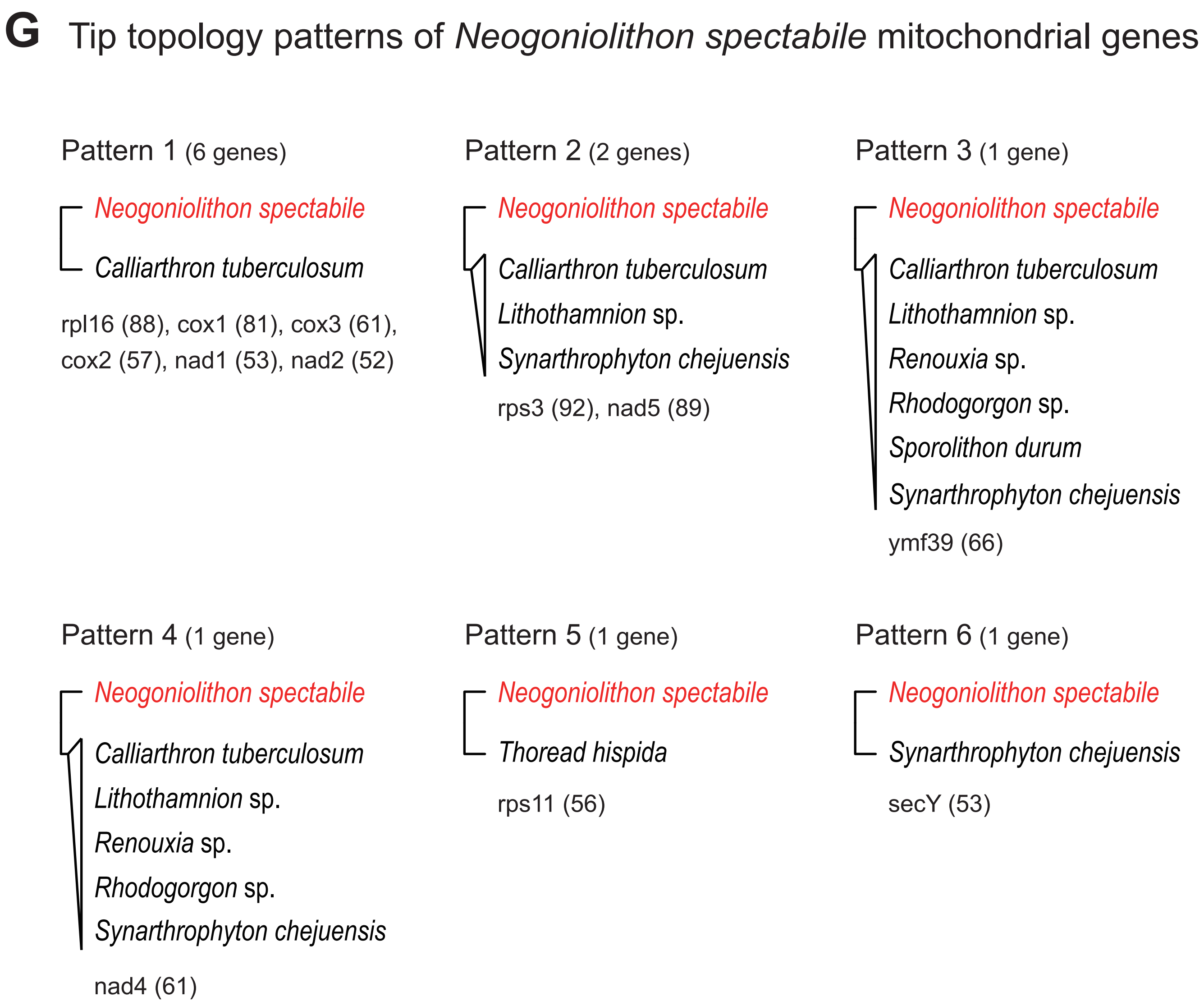

Supplementary fig. S8. The tip topology patterns of mitochondrial gene phylogenies from 7 coralline species. (*G*) Tip topology patterns of *Neogoniolithon spectabile*.

H

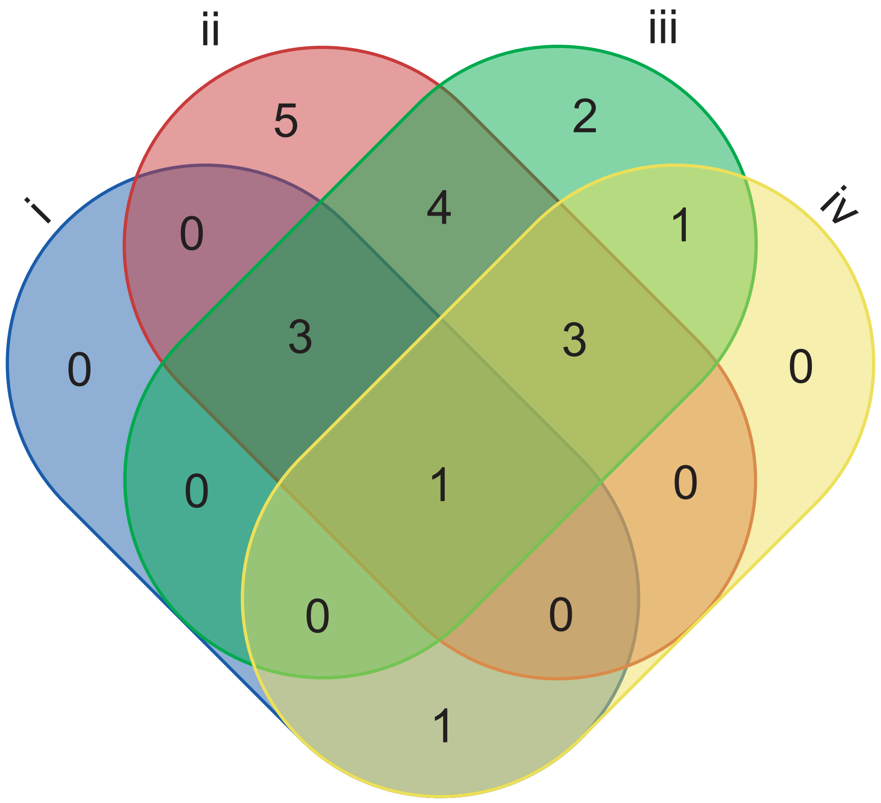

Groups	Number of genes	Genes
i, ii, iii, iv	1	nad1
i, ii, iii	3	nad5, nad4, rps3
ii, iii, iv	3	nad2, cox1, cox3
i, iv	1	rpl16
ii, iii	4	sdh2, nad3, ymf39, rps11
iii, iv	1	cox2
ii	5	rpl1, atp8, atp6, cob, rps12
iii	2	nad6, sdh4

Supplementary fig. S8. The tip topology patterns of mitochondrial gene phylogenies from 7 coralline species. (*H*) Venn diagram of number of genes in tip topology patterns (i ~ iv in fig. 2B) and their gene lists.

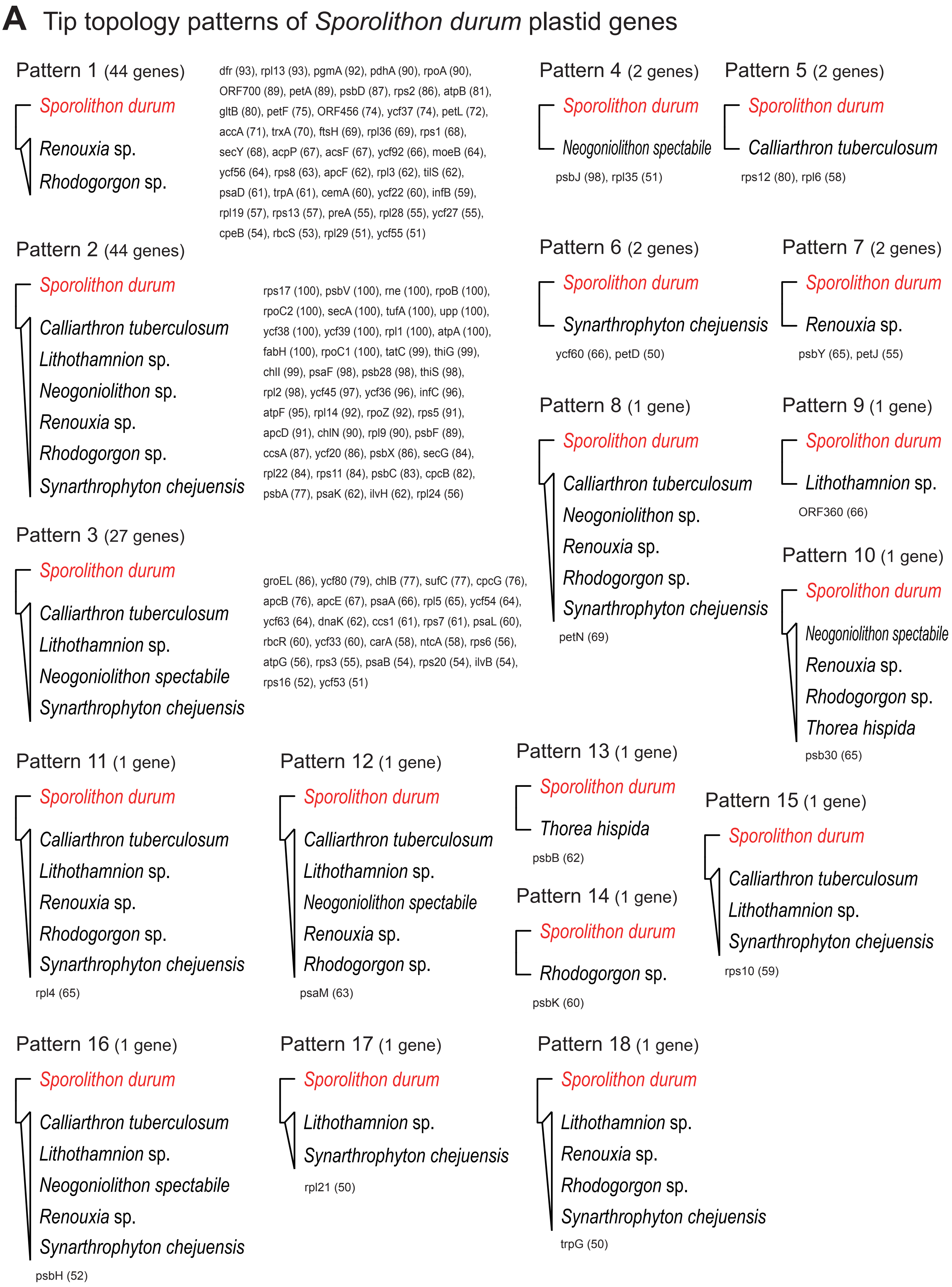

Supplementary fig. S9. The tip topology patterns of plastid gene phylogenies from 7 coralline species. (*A*) Tip topology patterns of *Sporolithon durum*.

B Tip topology patterns of *Renouxia* sp. plastid genes

Pattern 1 (170 genes)

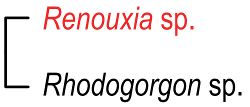

ORF456 (100), ORF700 (100), apcF (100), ccs1 (100), ccsA (100), cemA (100), chlI (100), dfr (100), ftsH (100), gltB (100), groEL (100), hisS (100), ilvB (100), infB (100), petA (100), pgmA (100), preA (100), psaA (100), psaB (100), rbcL (100), rpoA (100), rpoB (100), rpoC2 (100), rps17 (100), rps1 (100), secA (100), secY (100), sufB (100), syfB (100), tatC (100), ycf38 (100), ycf39 (100), acsF (99), apcE (99), atpA (99), atpF (99), atpG (99), chlB (99), ilvH (99), psbC (99), rne (99), rpl1 (99), rpl2 (99), rpl4 (99), rps14 (99), ycf21 (99), ycf55 (99), ycf56 (99), atpD (98), carA (98), ftrB (98), psaF (98), rpl22 (98), rpl24 (98), rpl5 (98), rps5 (98), rps7 (98), tilS (98), trpG (98), ycf4 (98), ycf92 (98), ORF360 (97), dnaK (97), fabH (97), pdhA (97), petL (97), rpl18 (97), rpl19 (97), rps19 (97), ycf37 (97), ycf58 (97), ycf80 (97), apcB (96), atpI (96), chlN (96), psbA (96), rpl3 (96), rps4 (96), sufC (96), ycf20 (96), ycf22 (96), ycf63 (96), ccdA (95), rpoC1 (95), rpoZ (95), upp (95), ycf45 (95), argB (94), atpB (94), clpC (94), rpl20 (94), rpl9 (94), rps12 (94), rps13 (94), trpA (94), ycf36 (94), accD (93), psb28 (93), secG (93), rbcS (92), rpl23 (92), rpl6 (92), accA (91), acpP (91), ycf27 (91), ycf65 (91), trxA (90), ycf3 (90), psbV (89), rps2 (89), thiG (89), ycf60 (89), ntcA (88), pbsA (88), rbcR (88), ORF65 (87), psaD (87), psaK (87), rps3 (87), ycf53 (87), chlL (86), rpl13 (86), rps6 (86), rps8 (86), ycf33 (86), ycf41 (86), accB (85), pdhB (85), tsf (85), cpcB (84), petF (84), psbB (84), rps10 (84), psaE (83), psbN (83), rpl35 (83), infC (81), rpl27 (80), rpl32 (80), rps11 (80), petB (79), ycf29 (79), rpl36 (78), petD (76), cpeB (75), psaI (75), apcD (73), apcA (72), bas1 (72), moeB (72), rps18 (72), atpE (71), cpcA (71), cbbX (70), rpl28 (70), cpcG (68), rps16 (67), rpl14 (66), petG (64), psbF (64), psbX (64), thiS (64), ycf52 (64), tufA (62), petN (61), rpl11 (61), ycf54 (59), rps20 (58), atpH (57), rpl29 (51)

Pattern 2 (2 genes)

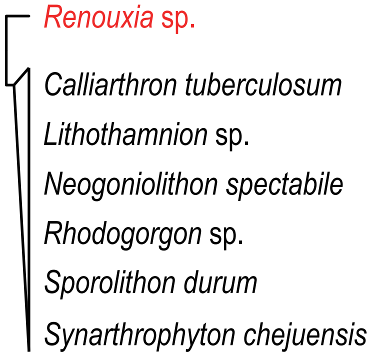

psaL (100), ycf19 (91)

Pattern 3 (2 genes)

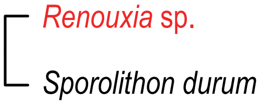

psbY (65), petJ (55)

Pattern 4 (2 genes)

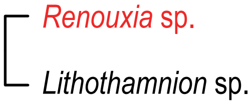

psaM (57), rpl33 (55)

Pattern 5 (1 gene)

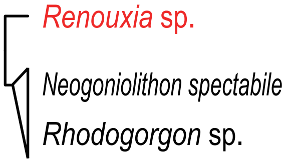

rpl16 (89)

Pattern 6 (1 gene)

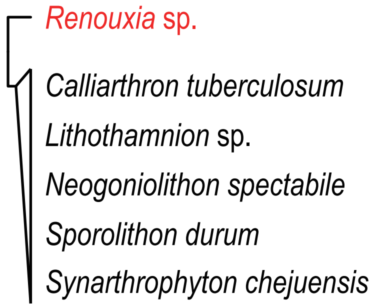

rpl21 (76)

Pattern 7 (1 gene)

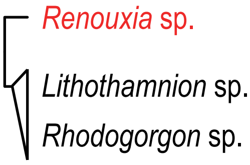

psbT (71)

Supplementary fig. S9. The tip topology patterns of plastid gene phylogenies from 7 coralline species. (*B*) Tip topology patterns of *Renouxia* sp.

C Tip topology patterns of *Rhodogorgon* sp. plastid genes

Pattern 1 (170 genes)

Rhodogorgon sp.
Renouxia sp.

ORF456 (100), ORF700 (100), apcF (100), ccs1 (100), ccsA (100), cemA (100), chlI (100), dfr (100), ftsH (100), gltB (100), groEL (100), hisS (100), ilvB (100), infB (100), petA (100), pgmA (100), preA (100), psaA (100), psaB (100), rbcL (100), rpoA (100), rpoB (100), rpoC2 (100), rps17 (100), rps1 (100), secA (100), secY (100), sufB (100), syfB (100), tatC (100), ycf38 (100), ycf39 (100), acsF (99), apcE (99), atpA (99), atpF (99), atpG (99), chlB (99), ilvH (99), psbC (99), rne (99), rpl1 (99), rpl2 (99), rpl4 (99), rps14 (99), ycf21 (99), ycf55 (99), ycf56 (99), atpD (98), carA (98), ftrB (98), psaF (98), rpl22 (98), rpl24 (98), rpl5 (98), rps5 (98), rps7 (98), tilS (98), trpG (98), ycf4 (98), ycf92 (98), ORF360 (97), dnaK (97), fabH (97), pdhA (97), petL (97), rpl18 (97), rpl19 (97), rps19 (97), ycf37 (97), ycf58 (97), ycf80 (97), apcB (96), atpI (96), chlN (96), psbA (96), rpl3 (96), rps4 (96), sufC (96), ycf20 (96), ycf22 (96), ycf63 (96), ccdA (95), rpoC1 (95), rpoZ (95), upp (95), ycf45 (95), argB (94), atpB (94), clpC (94), rpl20 (94), rpl9 (94), rps12 (94), rps13 (94), trpA (94), ycf36 (94), accD (93), psb28 (93), secG (93), rbcS (92), rpl23 (92), rpl6 (92), accA (91), acpP (91), ycf27 (91), ycf65 (91), trxA (90), ycf3 (90), psbV (89), rps2 (89), thiG (89), ycf60 (89), ntcA (88), pbsA (88), rbcR (88), ORF65 (87), psaD (87), psaK (87), rps3 (87), ycf53 (87), chlL (86), rpl13 (86), rps6 (86), rps8 (86), ycf33 (86), ycf41 (86), accB (85), pdhB (85), tsf (85), cpcB (84), petF (84), psbB (84), rps10 (84), psaE (83), psbN (83), rpl35 (83), infC (81), rpl27 (80), rpl32 (80), rps11 (80), petB (79), ycf29 (79), rpl36 (78), petD (76), cpeB (75), psaI (75), apcD (73), apcA (72), bas1 (72), moeB (72), rps18 (72), atpE (71), cpcA (71), cbbX (70), rpl28 (70), cpcG (68), rps16 (67), rpl14 (66), petG (64), psbF (64), psbX (64), thiS (64), ycf52 (64), tufA (62), petN (61), rpl11 (61), ycf54 (59), rps20 (58), atpH (57), rpl29 (51)

Pattern 2 (3 genes)

Rhodogorgon sp.
Calliarthron tuberculosum
Lithothamnion sp.
Neogoniolithon spectabile
Renouxia sp.
Sporolithon durum
Synarthrophyton chejuensis

rpl21 (90), nblA (83), psaJ (54)

Pattern 3 (2 genes)

Rhodogorgon sp.
Neogoniolithon spectabile

rpl16 (62), rps9 (52)

Pattern 4 (1 gene)

Rhodogorgon sp.
Calliarthron tuberculosum
Lithothamnion sp.
Neogoniolithon spectabile
Sporolithon durum
Synarthrophyton chejuensis

psaL (76)

Pattern 5 (1 gene)

Rhodogorgon sp.
Thorea hispida

psbJ (75)

Pattern 6 (1 gene)

Rhodogorgon sp.
Neogoniolithon spectabile
Thorea hispida

psb30 (66)

Pattern 7 (1 gene)

Rhodogorgon sp.
Sporolithon durum

psbK (60)

Pattern 8 (1 gene)

Rhodogorgon sp.
Lithothamnion sp.
Renouxia sp.

psaM (59)

Supplementary fig. S9. The tip topology patterns of plastid gene phylogenies from 7 coralline species. (*C*) Tip topology patterns of *Rhodogorgon* sp.

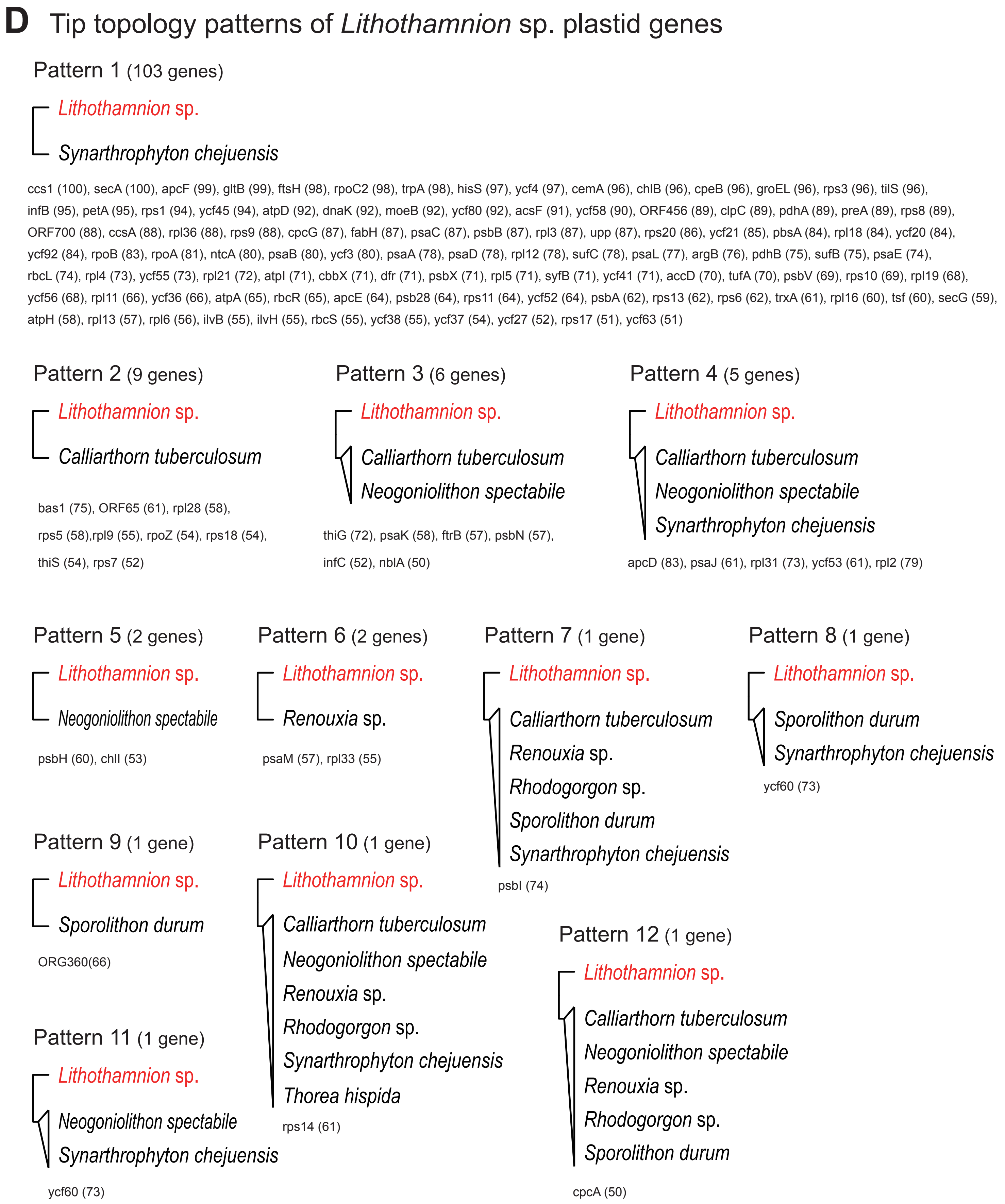

Supplementary fig. S9. The tip topology patterns of plastid gene phylogenies from 7 coralline species. (*D*) Tip topology patterns of *Lithothamnion* sp.

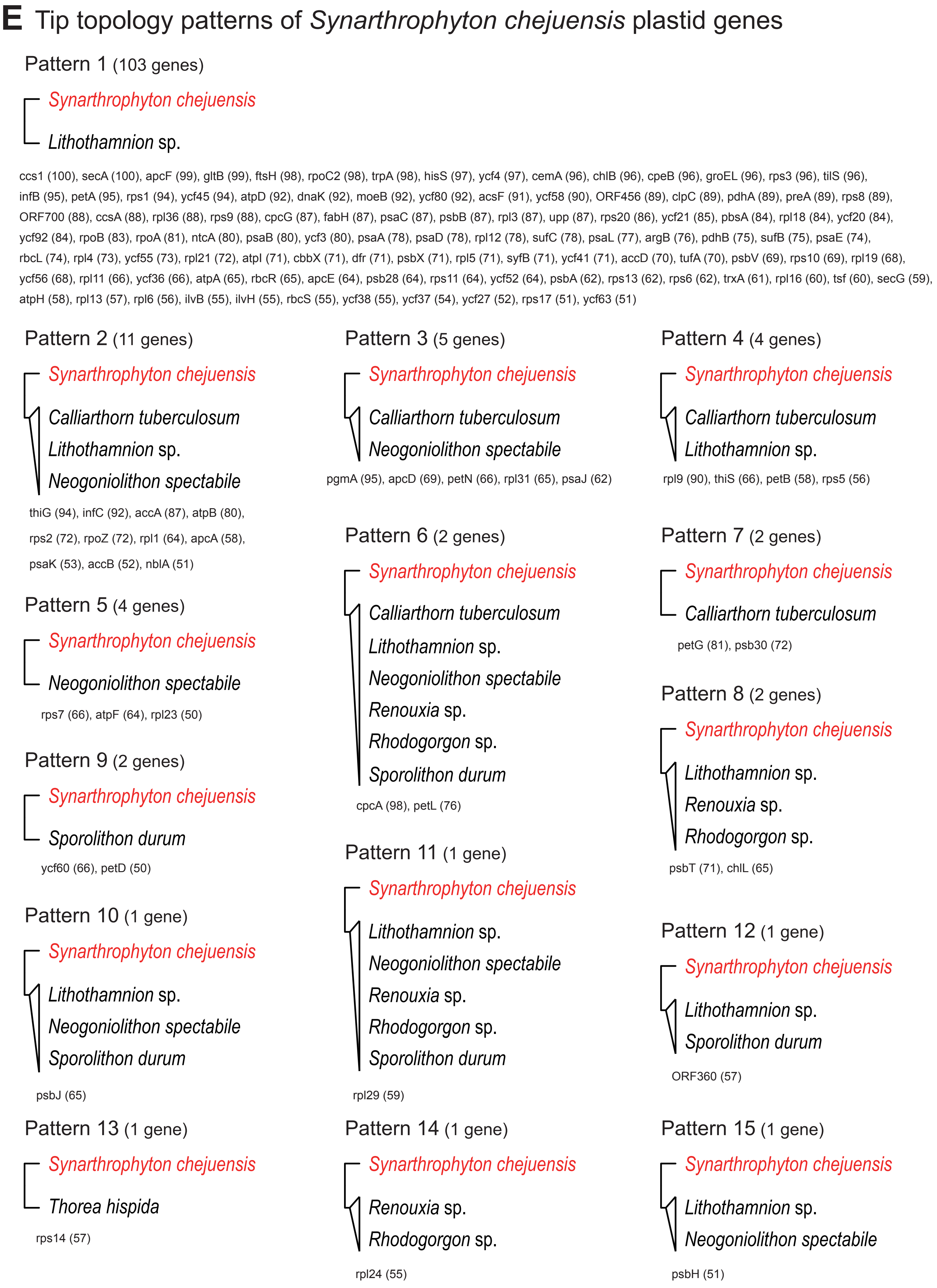

Supplementary fig. S9. The tip topology patterns of plastid gene phylogenies from 7 coralline species. (*E*) Tip topology patterns of *Synarthrophyton chejuensis*.

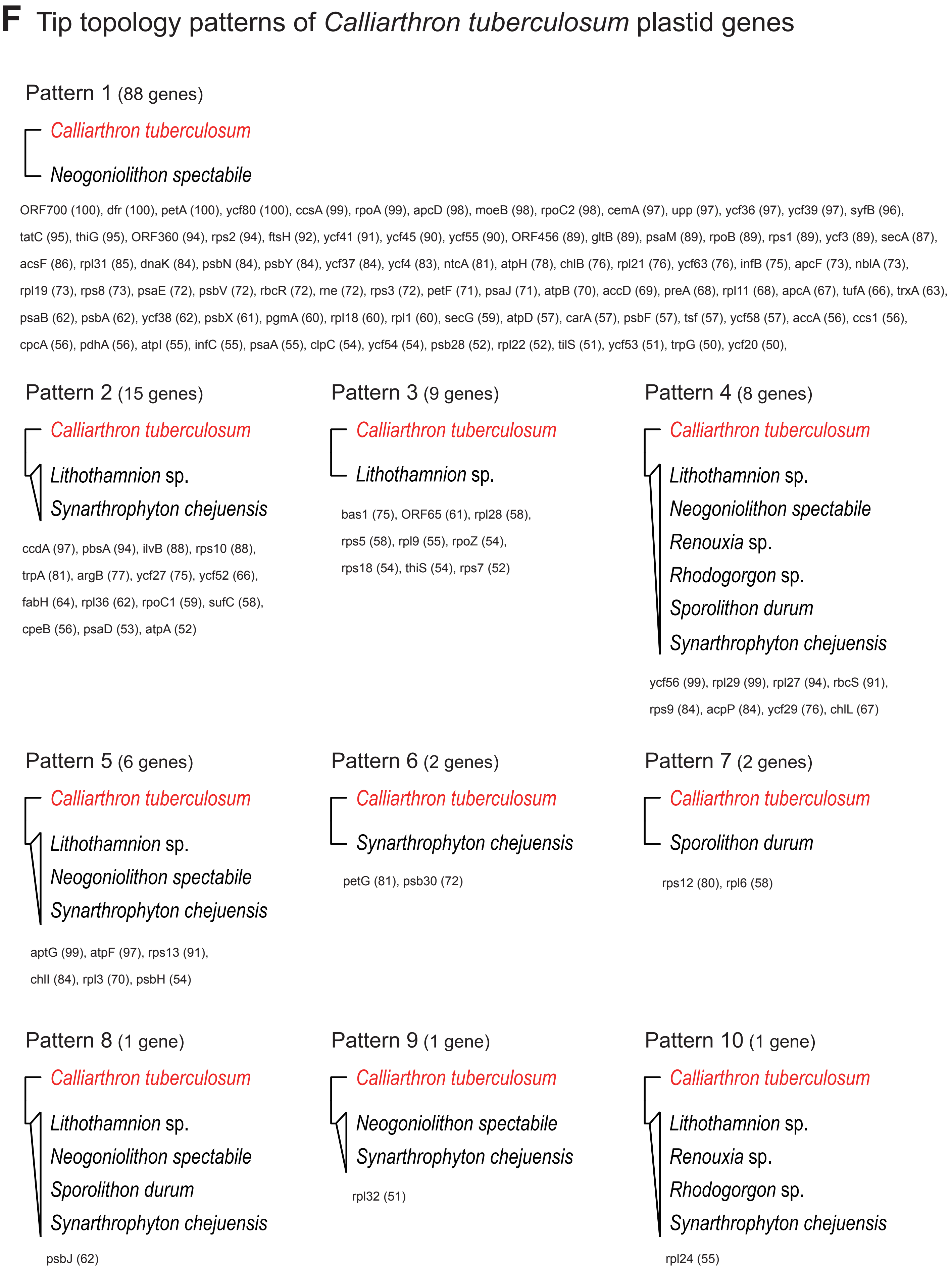

Supplementary fig. S9. The tip topology patterns of plastid gene phylogenies from 7 coralline species. (*F*) Tip topology patterns of *Calliarthron tuberculosum*.

G Tip topology patterns of *Neogoniolithon spectabile* plastid genes

Pattern 1 (88 genes)

Neogoniolithon spectabile
Calliarthron tuberculosum

ORF700 (100), dfr (100), petA (100), ycf80 (100), ccsA (99), rpoA (99), apcD (98), moeB (98), rpoC2 (98), cemA (97), upp (97), ycf36 (97), ycf39 (97), syfB (96), tatC (95), thiG (95), ORF360 (94), rps2 (94), ftsH (92), ycf41 (91), ycf45 (90), ycf55 (90), ORF456 (89), gltB (89), psaM (89), rpoB (89), rps1 (89), ycf3 (89), secA (87), acsF (86), rpl31 (85), dnaK (84), psbN (84), psbY (84), ycf37 (84), ycf4 (83), ntcA (81), atpH (78), chlB (76), rpl21 (76), ycf63 (76), infB (75), apcF (73), nblA (73), rpl19 (73), rps8 (73), psaE (72), psbV (72), rbcR (72), rne (72), rps3 (72), petF (71), psaJ (71), atpB (70), accD (69), preA (68), rpl11 (68), apcA (67), tufA (66), trxA (63), psaB (62), psbA (62), ycf38 (62), psbX (61), pgmA (60), rpl18 (60), rpl1 (60), secG (59), atpD (57), carA (57), psbF (57), tsf (57), ycf58 (57), accA (56), ccs1 (56), cpcA (56), pdhA (56), atpI (55), infC (55), psaA (55), clpC (54), ycf54 (54), psb28 (52), rpl22 (52), tilS (51), ycf53 (51), trpG (50), ycf20 (50),

Pattern 2 (19 genes)

Neogoniolithon spectabile
Calliarthron tuberculosum
Lithothamnion sp.
Synarthrophyton chejuensis

apcE (100), cpeB (99), cpcG (98), ilvB (97), rpl36 (97), rpoC1 (93), ycf52 (92), rpl14 (90), apcB (85), atpA (84), rps16 (73), rpl20 (72), rpl9 (68), thiS (68), trpA (62), fabH (61), ycf92 (56), rps5 (54), cpcB (54)

Pattern 3 (12 genes)

Neogoniolithon spectabile
Calliarthron tuberculosum
Lithothamnion sp.
Renouxia sp.
Rhodogorgon sp.
Sporolithon durum
Synarthrophyton chejuensis

argB (99), rpl4 (97), psaD (91), petD (89), psbZ (88), rpl33 (87), ORF65 (81), rps12 (78), psbI (74), psbK (67), ycf65 (53), pdhB (50),

Pattern 4 (3 genes)

Neogoniolithon spectabile
Synarthrophyton chejuensis

rps7 (66), atpF (64), rpl23 (50)

Pattern 5 (2 genes)

Neogoniolithon spectabile
Calliarthron tuberculosum
Lithothamnion sp.
Renouxia sp.
Rhodogorgon sp.
Synarthrophyton chejuensis

hisS (100), ycf21 (90)

Pattern 6 (2 genes)

Neogoniolithon spectabile
Sporolithon durum

psbJ (98), rpl35 (51)

Pattern 7 (2 genes)

Neogoniolithon spectabile
Thoread hispida

psb30 (73), rpl28 (60)

Pattern 8 (2 genes)

Neogoniolithon spectabile
Rhodogorgon sp.

rpl16 (62), rps9 (52)

Pattern 9 (2 genes)

Neogoniolithon spectabile
Lithothamnion sp.

psbH (60), chlI (53)

Pattern 10 (2 genes)

Neogoniolithon spectabile
Renouxia sp.
Rhodogorgon sp.

bas1 (59), rpl6 (53)

Pattern 11 (1 gene)

Neogoniolithon spectabile
Calliarthron tuberculosum
Lithothamnion sp.
Sporolithon durum
Synarthrophyton chejuensis

rps10 (74)

Pattern 12 (1 gene)

Neogoniolithon spectabile
Calliarthron tuberculosum
Renouxia sp.
Rhodogorgon sp.
Sporolithon durum

psbE (65)

Supplementary fig. S9. The tip topology patterns of plastid gene phylogenies from 7 coralline species. (*G*) Tip topology patterns of *Neogoniolithon spectabile*.

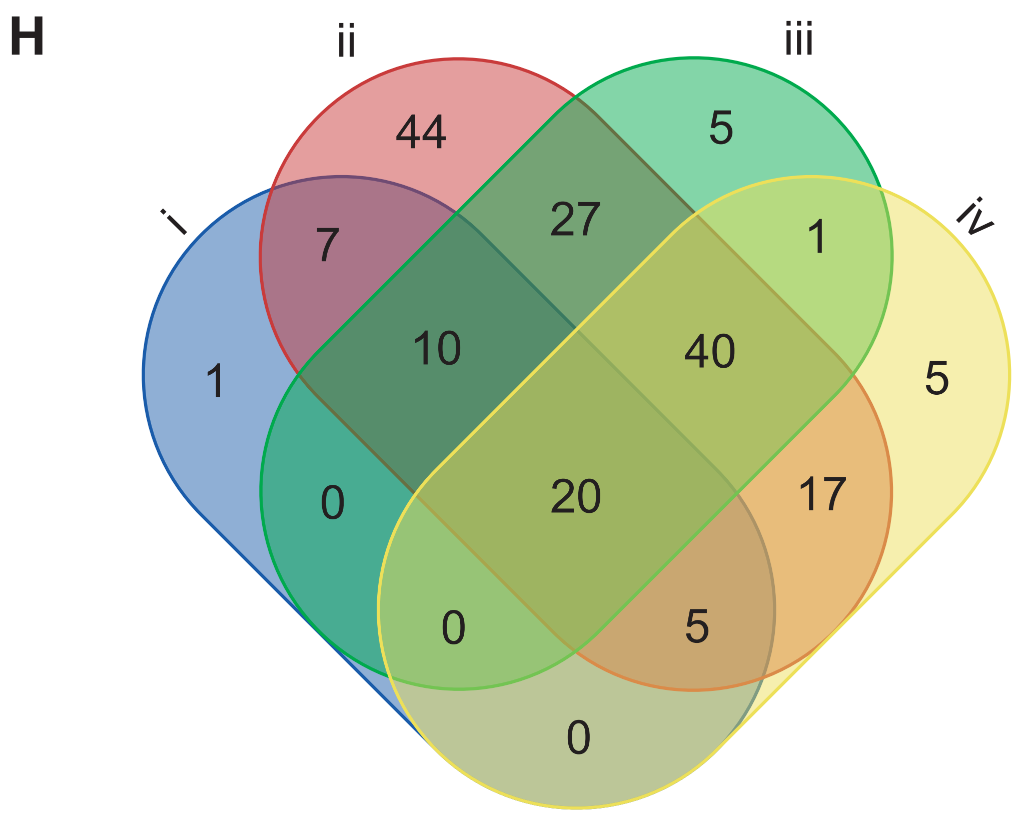

Groups	Number of genes	Genes
i, ii, iii, iv	20	infB, cemA, petA, preA, orf456, tilS, dfr, trxA, ftsh, rps1, pdhA, rpoA, ycf37, orf700, apcF, acsF, gltB, rps8, ycf55, moeB
i, ii, iii	10	rpl36, rbcS, ycf92, cpeB, psaD, ycf27, ycf56, rpl13, trpA, rps13
i, ii, iv	5	rps2, pgmA, atpB, accA, petF
ii, iii, iv	40	psaA, syfB, atpD, rpl18, chlB, ccs1, ycf41, clpC, tufA, ycf80, secA, ycf3, ycf63, psbA, atpI, psbX, rpl19, psb28, ycf45, dnaK, rps3, rpoB, ntcA, psaB, rpl11, rbcR, upp, psaE, ccsA, ycf20, ycf38, ycf36, ycf58, secG, accD, atpH, rpoC2, psbV, tsf, ycf4
i, ii	7	secY, rpl28, ycf22, rpl29, rpl9, petL, acpP
ii, iii	27	rpl5, pdhB, sufB, rps6, ilvB, fabH, rps11, rpl3, hisS, ycf52, cbbX, rpl4, rpl6, ycf21, rps10, pbsA, apcE, argB, psbB, rps20, rbcL, rps17, groEL, atpA, ilvH, sufC, cpcG
ii, iv	17	thiG, ycf53, apcA, carA, rpl1, ycf54, rpl22, ycf39, cpcA, infC, psbN, trpG, orf360, psbF, tatC, apcD, rne
iii, iv	1	rpl21
i	1	psbD
ii	44	rpl20, ycf65, ycf60, petN, psaK, atpE, rps4, petG, rps18, chlL, rpl23, petD, rpl32, rpl14, chlN, atpG, rps16, rpl27, rps14, rpoC1, psbC, psaF, orf65, rps7, ycf33, atpF, rpoZ, apcB, rps12, rpl2, psaI, rps5, accB, rpl35, petB, rps19, ycf29, ccdA, ftrB, thiS, cpcB, chlI, bas1, rpl24
iii	5	rpl16, rps9, rpl12, psaL, psaC
iv	5	nblA, psaJ, rpl31, psaM, psbY

Supplementary fig. S9. The tip topology patterns of plastid gene phylogenies from 7 coralline species. (*H*) Venn diagram of number of genes in tip topology patterns (i ~ iv in fig. 2B) and their gene lists.

A Mitochondrial genes

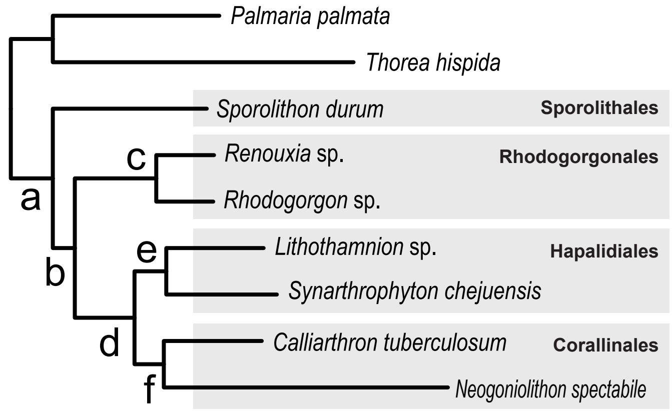

sub-sampling size (TIGER program)

	5,989 (100%)	3,604 (60%)	2,811 (46%)	1,985(AA) (33%)
a	100	100	100	100
b	93	96	83	61
c	100	100	100	100
d	100	100	100	100
e	100	100	100	100
f	90	95	93	93

B Plastid genes

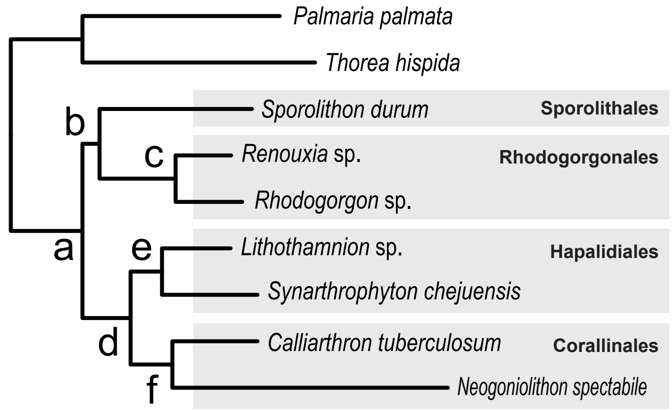

	49,710 (100%)	26,630 (53%)	21,110 (42%)	19,274*(AA) (38%)
a	100	100	100	100
b	98	95	72	57
c	100	100	100	100
d	100	100	100	100
e	100	100	100	100
f	100	100	100	100

* Topology was changed to mitochondrial tree

Supplementary fig. S10. The sub-sampled datset-based phylogenies of concatenated alignments of amino acid sequences in mitochondria and plastid genes by the TIGER program (option: -b 50; Cummins and McInerney 2011). (*A*) Distirbutions of bootstrap supporting values in each divergence point of concatenated mitochondria phylogeny between original and each sub-sampled datasets. (*B*) Distirbutions of bootstrap supporting values in each divergence point of concatenated plastid phylogeny between original and each sub-sampled datasets. Asterisk (*) indicates concatenated mitochondria tree topology in a sub-sampled dataset of plastid genes.

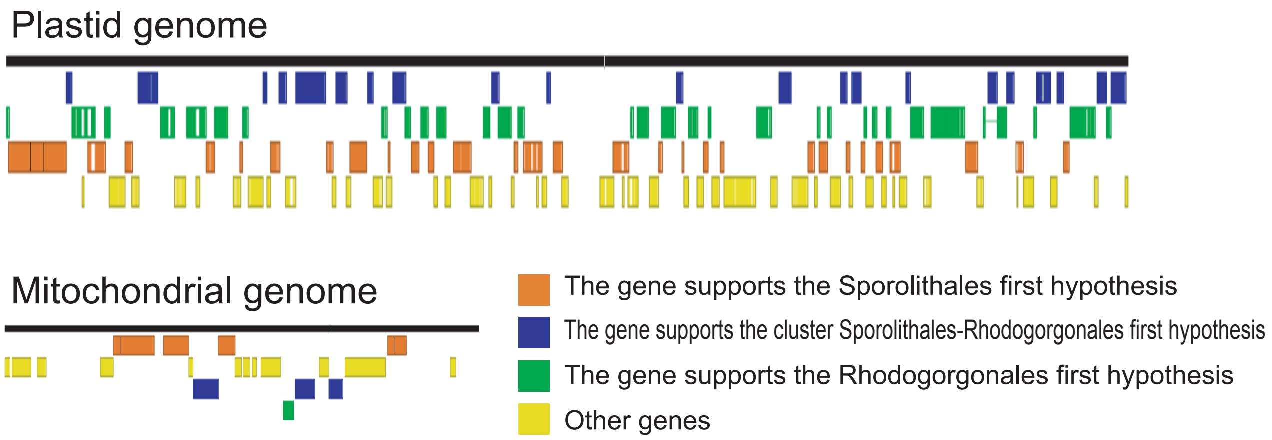

Supplementary fig. S11. Schematic distrubtion of organelle genes depends on the three evolutionary histories in coralline algae.

A **Non-synonymous substitution rate (dN) using all types of mitochondrial (MT) genes**

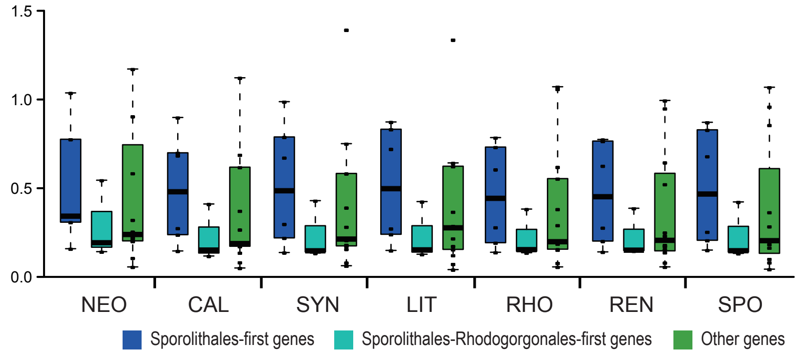

B **Non-synonymous substitution rate (dN) using all types of plastid (PT) genes**

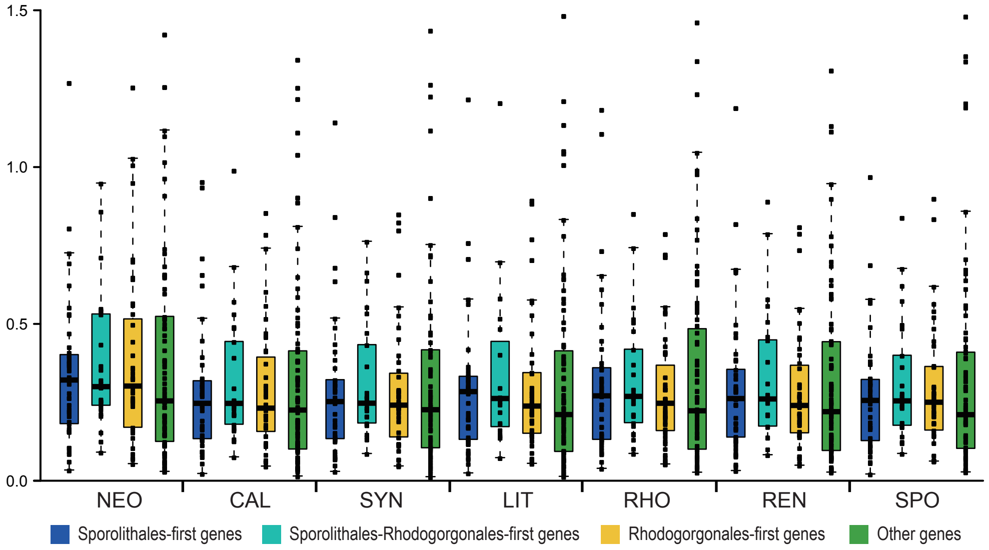

Supplementary fig. S12. Non-synonymous substitution rate (dN) using all types of organelle genes between *Palmaria palmate* (outgroup taxa) and coralline species. (*A*) The boxplots of dN values using coralline mitochondrial genes. (*B*) The boxplots of dN values using plastid genes. Abbreviations: NEO = *Neogoniolithon spectabile*, CAL = *Calliarthron tuberculosum*, SYN = *Synarthrophyton chejuensis*, LIT = *Lithothamnion* sp., RHO = *Rhodogorgon* sp., REN = *Renouxia* sp., and SPO = *Sporolithon durum*.
